# A Switchable Palladium(II) Trefoil Entangled Tetrahedron with Temperature Dependence and Concentration Independence

**DOI:** 10.1002/anie.202210476

**Published:** 2022-08-23

**Authors:** Jess L. Algar, James A. Findlay, Jack D. Evans, Dan Preston

**Affiliations:** ^1^ Research School of Chemistry Australian National University Canberra ACT 2600 Australia; ^2^ Centre for Advanced Nanomaterials and Department of Chemistry The University of Adelaide Adelaide SA 5000 Australia

**Keywords:** Interlocked Architecture, Palladium, Self-Assembly

## Abstract

Self‐assembly makes metallo‐interlocked architectures attractive targets, but being in equilibrium with smaller species means that they can suffer from dilution effects. We show that a junctioned system gives rise to a [Pd_4_(L)_2_]^8+^ trefoil entangled tetrahedron irrespective of concentration. Heating the sample reversibly shifts the equilibrium from the knot to an isomeric non‐interlocked dual metallo‐cycle, demonstrating that thermodynamic equilibria can still be exploited for switching even in the absence of concentration effects.

## Graphical abstract

A junctioned system has been developed that gives rise to a [Pd_4_(L)_2_]^8+^ trefoil entangled tetrahedron irrespective of the concentration. Heating the sample reversibly shifts the equilibrium from the knot to an isomeric non‐interlocked dual metallocycle, thus demonstrating that thermodynamic equilibria can still be exploited for switching even in the absence of concentration effects.

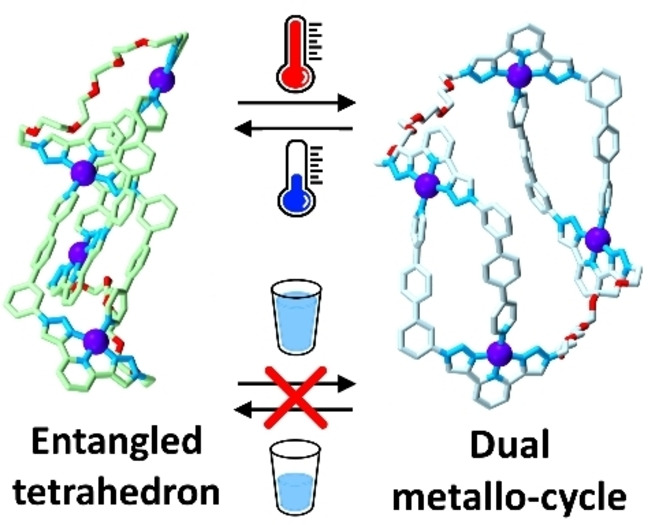

Interlocked architectures, including catenanes,^[1]^ rotaxanes,^[2]^ Borromean rings^[3]^ and various types of knots,^[4]^ provide routes to complex topologies similar to those that give natural molecular machinery such as proteins their exquisite specificity and control. Emergent applications for interlocked architectures have been demonstrated in areas including drug release,^[5]^ sensing,^[6]^ muscle‐like movement,^[7]^ and catalysis.^[8]^ Many of these applications revolve around the capacity of the interlocked architecture to alter its conformation in response to stimuli, in the same way that protein activity is regulated by allostery.

Metallo‐interlocked architectures have likewise been reported in a wide variety of topologies.^[9]^ A variety of these that are considered exceedingly rare are branched or “junctioned” assemblies.^[10]^ The first molecular example (a universal‐3‐ravel) was reported in the solid state by Lindoy and co‐workers featuring Fe^III^ and *bis*‐diketonate ligands.^[11]^ Wu and co‐workers have recently reported an anion‐templated hydrogen‐bonded ravel.^[12]^ Fujita and co‐workers have also reported examples of entangled architectures with Ag^I^ and tripodal pyridyl ligands,^[13]^ and Nitschke and co‐workers have reported tetrahedra (assemblies with four 3‐way junctions) with unusual topologies.^[14]^ There is a renaissance of topologically complex metallo‐interlocked architectures at present, at least in part due to their relative ease of synthesis under thermodynamic control. Their reversible self‐assembly also allows for switching of these structures, normally between an enthalpically favoured species with more components and a smaller entropically favoured species, in contrast to a conformational change observed in irreversibly interlocked organic architectures. Alteration of concentration[^9c^, ^15^] and solvent[^15^, ^16^] have been employed as stimuli in this regard.

Concentration dependence in interlocking is potentially problematic for the development of many types of functional systems. Often switching from a high proportion of one species to a high proportion of the other would require concentration changes of many orders of magnitude. More concerningly, concentration dependence means that in dilute solutions, the system may exist with molecules effectively all in the smaller form, removing any switchability at all. Metallo‐interlocked structures formed with the ease of self‐assembly but with equilibria that exist independently of concentration would therefore be highly advantageous. This concentration dependence arises as a direct consequence of the equilibrium expression when the interlocked species is formed from more components than the smaller, non‐interlocked species.

We directly address this issue here, with a system which can be switched using temperature between an interlocked trefoil entangled tetrahedron and a non‐interlocked dual macrocycle (Figure 1 bottom), with the equilibrium existing independent of concentration. We used a core ligand scaffold consisting of a mono‐pyridyl arm and a tridentate site (Figure 1 top), analogous to our previously reported system that exhibited temperature‐ and concentration‐induced switching between metallo‐cyclic and catenated forms.^[17]^ That scaffold had 3+1 complementarity^[18]^ and so formed [Pd_2_L_2_]^4+^ macrocycles. The driving force for catenation in this previous work, like the vast majority of the examples given above as well as many other conformationally‐folded metallo‐structures,^[19]^ was π–π interactions (and potentially solvophobicity). In this current work, the tridentate site is *bis*‐1,2,3‐triazole‐2,6‐pyridine, and the formation of the second triazole allows for easy control over the identity of the substituent attached to the core, allowing us to tune the behaviour of the system.

**Figure 1:**
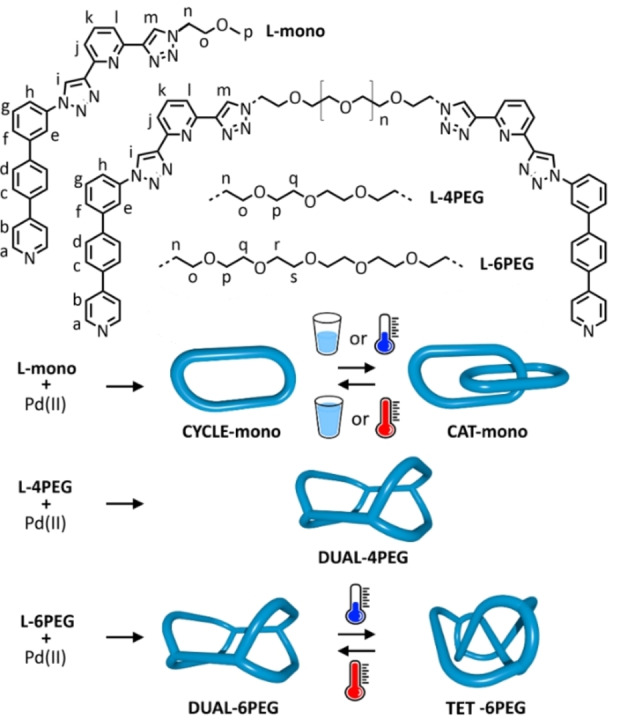
Top: core ligand scaffold used in this project, with the identity of the three ethylene glycol chains of the three ligands also given: **L‐mono** possesses a single core, while **L‐4PEG** and **L‐6PEG** have *two* cores bridged by 4PEG and 6PEG chains respectively. Bottom: identity and switchability of the **mono**, **4PEG** and **6PEG** systems used in this study.

We first synthesised the model ligand, **L‐mono**, with R=ethylene glycol monomethyl (Figure 1 top). The 2 : 2 combination of the ligand and [Pd(CH_3_CN)_4_](BF_4_)_2_ in deuterated dimethylsulfoxide ([D_6_]DMSO) at [**L‐mono**]=15 mM gave a ^1^H nuclear magnetic resonance (NMR) spectrum with a single set of resonances per ligand environment, indicating a highly symmetrical product (Figure 2a and b).

**Figure 2:**
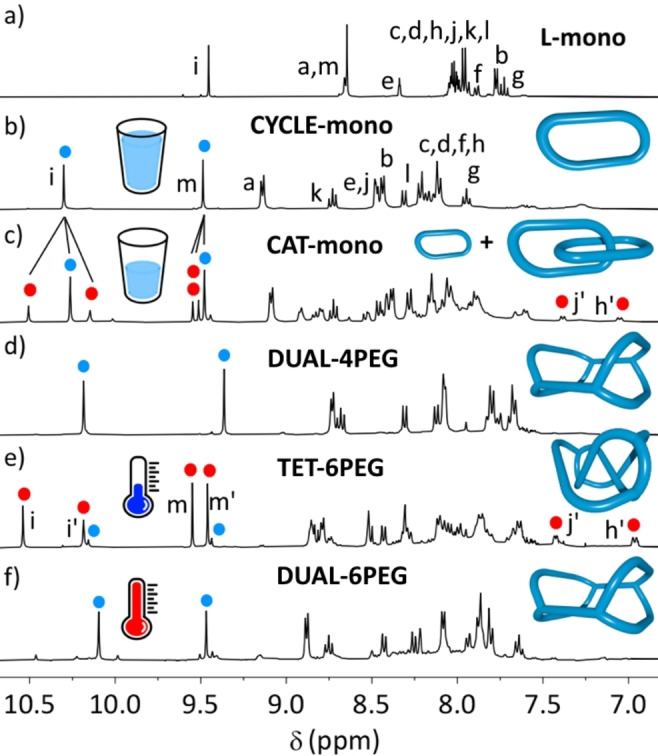
Partial stacked ^1^H NMR spectra (400 MHz, [D_6_]DMSO) at 298 K of a) **L‐mono**, b) 2 : 2 combination of **L‐mono** and Pd^II^ at 15 mM, giving **CYCLE‐mono**, c) 2 : 2 combination of **L‐mono** and Pd^II^ at 60 mM, giving a 1 : 3 ratio of **CAT‐mono**/**CYCLE‐mono**, d) 2 : 4 combination of **L‐4PEG** and Pd^II^, giving **DUAL‐4PEG**, e) 1 : 2 combination of **L‐6PEG** and Pd^II^, giving a 85 : 15 ratio of **TET‐6PEG**/**DUAL‐6PEG**, and at 361 K of f) 2 : 4 combination of **L‐6PEG** and Pd^II^, giving a 1 : 9 ratio of **TET‐6PEG**/**DUAL‐6PEG**. Blue spots are non‐interlocked species, red spots are interlocked.

Peaks were shifted downfield with respect to the free ligand, particularly for resonances associated with the tridentate site (e.g. triazole peaks H_i_ and H_m_, pyridyl peak H_k_) and monodentate site (pyridyl peaks H_a_ and H_b_), indicating coordination to the metal ion. High resolution electrospray ionisation mass spectrometry (HR ESI‐MS) of the sample confirmed identity as the metallocycle [Pd_2_(**L‐mono**)_2_]^4+^: **CYCLE‐mono** (e.g. *m*/*z*=303.5668 [**CYCLE‐mono**]^4+^, calc. 303.5560, Supporting Information). Molecular modelling (xtb‐GFN2,^[20]^ DMSO solvent field) confirmed that the 2+2 structural arrangement was feasible (Figure 3a).

**Figure 3:**
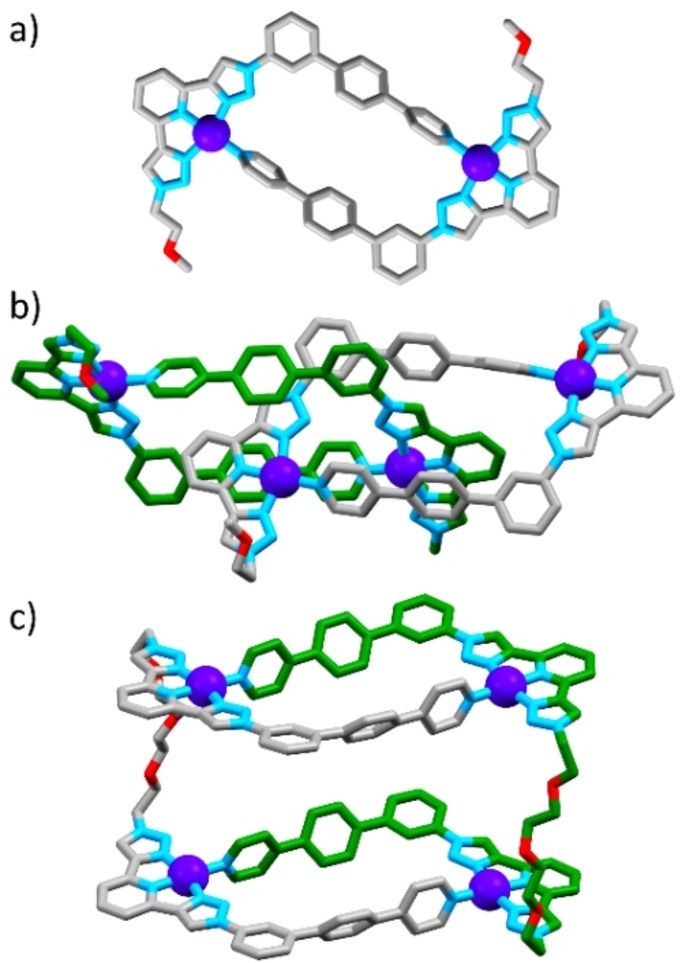
Representations of the molecular models of a) **CYCLE‐mono**, b) **CAT‐mono**, and c) **DUAL‐4PEG**. Structures in (a) and (c) were modelled using xtb‐GFN2^[20]^ while that in b is from DFT (BP86, def2‐SVP/def2‐TZVPP). Colours: carbon grey or green, nitrogen light blue, oxygen red, palladium purple, hydrogen atoms omitted for clarity.

Under more concentrated conditions, a second set of peaks with two resonances per environment began to grow into the ^1^H NMR spectra (Figure 2c, Supporting Information). The two sets of new peaks diffused at the same rate as each other in the ^1^H DOSY NMR spectrum (*D*=0.68×10^−10^ m^2^ s^−1^) and more slowly than **CYCLE‐mono** (*D*=0.83×10^−10^ m^2^ s^−1^). This is consistent with our previous study, and the work of many others, indicating catenation of two metallo‐cyclic units, [(Pd_2_(**L‐mono**)_2_)_2_]^8+^ (**CAT‐mono**). We exclude the possibility of a ring‐in‐ring structure:[^9d^, ^16^, ^21^] from the model of the metallocycle, the cavity is not large enough to contain another **CYCLE‐mono** metallacycle “edgeways” (Supporting Information). The presence of this species was confirmed via HR ESI‐MS (e.g. *m*/*z*=953.4899, [**CAT‐mono**+5 BF_4_]^3+^, Supporting Information). The two sets of environments in the ^1^H NMR spectrum correspond to the different ligand environments from each of the ligands in a single metallo‐cycle: ‘outer’ downfield while the “inner” is upfield as it is sequestered from the polar solvent and positioned to experience anisotropic effects from aromatic rings on either side. At [**L‐mono**]=60 mM at 298 K there was a 1 : 3 ratio between the **CYCLE‐mono** and the catenane (Supporting Information).

In addition to concentration dependence, the system was temperature dependent. Heating moved the equilibrium towards the non‐interlocked **CYCLE‐mono**. Analysis with a van't Hoff plot revealed that the conversion of **CYCLE‐mono** to **CAT‐mono** was enthalpically favoured and entropically disfavoured (Δ*H*=−75.3 kJ mol^−1^, Δ*S*=−227 J K^−1^ mol^−1^, Supporting Information), relatively similar numbers to our original study. We were unable to crystallise the catenane, but density functional theory (DFT) calculations (BP86,^[22]^ def2‐SVP for C, H, def2‐TZVPP for all other atoms^[23]^) were employed to provide structural insight. The optimised structure (Figure 3b) was consistent with the 2D NOESY data which showed correlation between resonances from the different sets of proton environments. Chiefly, there was a correlation between the resonance of the phenyl proton directed into the cavity of the metallo‐cycle in the “outer” ligand environment (e), and the “inner” triazole resonance (i′). From the DFT structure in this energetically preferred and experimentally confirmed conformation, the distance between “inner” and “outer” methylene carbons of the ethylene glycol chain adjacent to the triazole was ≈14.5 Å.

With this information in hand, we envisaged ligands in which two core ligand scaffolds were joined together through a polyethylene glycol linker (Figure 1 top). The length of this linker would define the maximum possible distance between the two tridentate sites. Considering the preferred conformation in **CAT‐mono**, initial modelling suggested that a hexaethylene glycol linker would span this distance in an interlocked system, while a tetraethylene glycol linker would not.

We therefore synthesised two more ligands with these linker lengths: **L‐6PEG** and **L‐4PEG** (Figure 1 top). We investigated the shorter‐linked **L‐4PEG** first, in a 2 : 4 combination between ligand and Pd^II^. The ^1^H NMR spectrum revealed a product with a single set of resonances that were downfield‐shifted with respect to the “free” ligand in environments close to the tridentate and monodentate sites, so interlocking had not occurred (Figure 2d). HR ESI‐MS analysis revealed the expected [Pd_4_(**L‐4PEG**)_2_]^8+^ species (e.g. *m*/*z*=425.0773, [Pd_4_(**L‐4PEG**)_2_+2 F^−^]^6+^), consistent with two metallo‐cycles linked together with the 4‐PEG chains into a dual metallo‐cyclic structure (**DUAL‐4PEG**, molecular model shown in Figure 3c).

Next we turned our attention to the longer‐linked ligand, **L‐6PEG**. The 2 : 4 combination of this ligand at 298 K with Pd^II^ gave a ^1^H NMR spectrum with 85 % predominance of a species with two resonances per ligand environment, shifted downfield and upfield with respect to one another. Several resonances experienced significant upfield shifting, in particular for the “inner” environment. For example, in comparison to the same peak in the non‐interlocked **DUAL‐4PEG**, the resonance of proton h′ was ≈0.85 ppm upfield, while j′ was 0.88 ppm upfield.

HR ESI‐MS confirmed the [Pd_4_(**L‐6PEG**)_2_]^8+^ identity of the compound with a series of 2+ to 5+ peaks differing in the number of associated BF_4_^−^ counterions (e.g. *m*/*z* **=**1040.8655 [Pd_4_(**L‐6PEG**)_2_+5BF_4_^−^]^3+^, Supporting Information). Via 2D NOESY spectroscopy, the connectivity of the two environments was conclusively evident (see below). Interlocking in the case of junctioned ligands by necessity results in knotting rather than catenation, and the formation of a trefoil entangled tetrahedron^[10b]^ (**TET‐6PEG**, Figure 4a). The 6PEG chain is not long enough to span between the two “outer” environments of the species, and hence **TET‐6PEG** consists of each **L‐6PEG** ligand having one half of the ligand in an “outer” environment, and the other in the “inner”.

**Figure 4:**
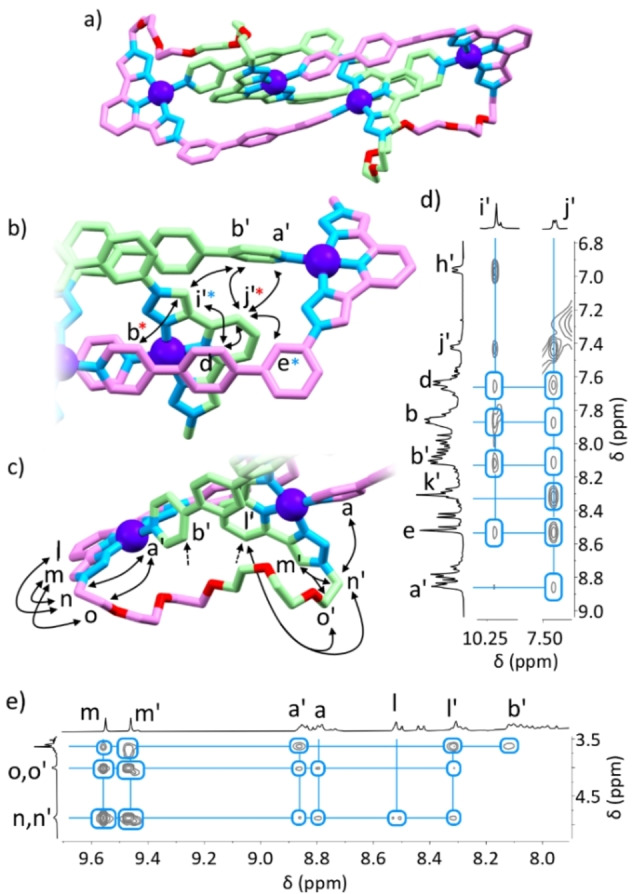
a) DFT structure of **TET‐6PEG** (BP86, def2‐SVP/def2‐TZVPP), with views showing b) “inner” and “outer” aromatic regions, c) alkyl and aromatic regions, and 2D ^1^H NOE correlations (400 MHz, [D_6_]DMSO, 298 K, 200 ms) between d) “inner” and “outer” aromatic environments and e) aromatic and alkyl environments. Correlations denoted through double headed arrows or asterisks of the same colour, and are between hydrogen atoms on the labelled carbon atoms. 2D NMR spectra in Supporting Information. Colours: carbon pink (outer) or green (inner), nitrogen light blue, oxygen red, palladium purple, hydrogen atoms omitted for clarity.

Heating the sample resulted in reduction in size of the peaks of the interlocked species and growth of the minor species (Figure 2f). At 361 K, the ratio of the species had changed from 85 : 15 to 1 : 9. This process was fully reversible. The new species had only a single set of resonances per environment, and the spectrum closely resembled that of **CYCLE‐mono** and **DUAL‐4PEG**, and we assign the identity of this species as **DUAL‐6PEG**. We were able to again extract the thermodynamic parameters for the system: Δ*H*=−55.1 kJ mol^−1^ and Δ*S*=−167 J K^−1^ mol^−1^, (Supporting Information). Comparison of these numbers to those from the **CYCLE‐mono**/**CAT‐mono** system is informative: interlocking is less enthalpically favoured than in **CAT‐mono**, presumably due to the tethered molecule having less capacity to adopt an ideal interlocked conformation, or more bond torsion and strain. And while **TET‐6PEG** is less relatively entropically disfavoured than **CAT‐mono**, interlocking still carries an entropic cost, presumably due to loss of translational and rotational freedom.

The interlocking process was solvent dependent: the introduction of less‐polar acetonitrile resulted in shifting of the equilibrium towards **DUAL‐6PEG** (at 9 : 1 acetonitrile/DMSO, no interlocked species was observed in the NMR spectrum). DFT calculations (BP86,^[22]^ def2‐SVP for C, H, def2‐TZVPP for all other atoms^[23]^) of the **TET‐6PEG** structure in various different solvent fields (and gas phase) confirmed qualitatively that as the dielectric constant decreased, the relative energetic favourability of the interlocked species over the cycle decreased, likely due to combined effects of lowered screening of cationic charge on Pd^II^ ions, and smaller solvophobic effects. This solvent dependence meant that the possibility of crystallising the trefoil entangled tetrahedron was extremely low.

The calculated interlocked structure (Figure 4a, Supporting Information) was fully consistent with the solution‐phase data. Key NOE correlations between both ligand environments, and between these environments and the 6PEG linker, were mirrored in spatial proximity between hydrogen atoms in the modelled structure (Figure 4b–e, Supporting Information). Namely, there were multiple correlations between proton environments lining each cleft (e.g. pyridyl resonances a′, b, b′, phenyl resonances d′, and the phenyl singlet directed into the cleft e) and the “inner” resonances of part of the tridentate binding site (i′ and j′). As well, correlations between the 6PEG linker and various aromatic environments were also observed (Figure 4e).

To briefly recap, the characterisation data includes: 1) the confirmation of molecular composition through HR ESI‐MS, 2) the well‐established desymmetrisation of the ^1^H NMR spectrum into “inner” and “outer” environments, 3) one of these environments being upfield shifted consistent with more shielding for the “inner” environment, 4) NOE correlations confirming that the two environments are part of a single, unified structure, and 5) the NOE correlations being fully consistent with the DFT‐calculated structure. Taken together, these data are only consistent with the formation of a branched knotted structure.

Importantly, the formation of **TET‐6PEG** was independent of concentration. ^1^H NMR spectra at a variety of concentrations from 25 mM (100 mM in terms of Pd^II^) to 0.0625 mM showed no variation in the proportion of **TET‐6PEG** to **DUAL‐6PEG** (Supporting Information).

While switching clearly involves the breaking and reformation of (coordination) bonds, unlike other switchable metallo‐interlocked systems **TET‐6PEG** and **DUAL‐6PEG** have the same molecular formula, and the same connectivity in terms of coordination bonds, meaning they are isomers of one another. This equivalence of composition is the source of the concentration‐independence of the system. Aside from the initial work from Lindoy and co‐workers,^[11]^ and the Fujita series of ravels,^[13]^ branched interlocked structures are few and far between. In our study, it is the junctioning and formation of a trefoil entangled tetrahedron rather than a catenane that circumvents concentration dependence, and junctions may therefore prove extremely useful for chemists seeking to create self‐assembled complex topologies that are switchable but do not suffer from dilution effects. There was a degree of serendipity in the position of the equilibrium in our system that meant that the relative proportion of interlocked to non‐interlocked species could be shifted from mostly one to mostly the other using temperature, but other junctioned systems could equally well exploit mechanisms such as chemical, acid/base or light stimuli. Accordingly, we expect these species to be increasingly targeted.

## Conflict of interest

The authors declare no conflict of interest.

## Supporting information

As a service to our authors and readers, this journal provides supporting information supplied by the authors. Such materials are peer reviewed and may be re‐organized for online delivery, but are not copy‐edited or typeset. Technical support issues arising from supporting information (other than missing files) should be addressed to the authors.

Supporting Information

## Data Availability

The data that support the findings of this study are available in the Supporting Information of this article.
