# Supplementary material for: A Switchable Palladium(II) Trefoil Entangled Tetrahedron with Temperature Dependence and Concentration Independence
Source: Angew Chem Int Ed Engl. 2022 Aug 23;61(39):e202210476. doi: 10.1002/anie.202210476 (PMC9805230; doi:10.1002/anie.202210476)
Supplement: Supplementary file 1 — Supporting Information [file ANIE-61-0-s001.pdf]

Angewandte Chemie
Eine Zeitschrift der Gesellschaft Deutscher Chemiker

Supporting Information

# A Switchable Palladium(II) Trefoil Entangled Tetrahedron with Temperature Dependence and Concentration Independence

*J. L. Algar, J. A. Findlay, J. D. Evans, D. Preston**

# Contents

1. Experimental ........ 3
  1.1. General ........ 3
  1.2. Precursors ........ 3
    1.2.1. 1 ........ 3
    1.2.2. 2 ........ 4
  1.3. Ligands ........ 5
    1.3.1. L-mono ........ 5
    1.3.2. L-4PEG ........ 7
  1.4. L-6PEG ........ 9
  1.5. Complexes ........ 10
    1.5.1. CYCLE-mono ........ 10
    1.5.2. CAT-mono ........ 14
    1.5.3. DUAL-4PEG ........ 18
    1.5.4. TET-6PEG ........ 21
    1.5.5. DUAL-6PEG ........ 25
2. DFT Calculations ........ 28
3. References ........ 29

# 1. Experimental

## 1.1. General

Unless otherwise stated, all reagents were purchased from commercial sources and used without further purification, except for:

2-(2-methyl-2'-hydroxy-1-butynyl)-6-(2-(trimethylsilyl)ethynyl)-pyridine[1]
Ethylene glycol monomethyl tosylate[2]
Tetraethylene glycol ditosylate[3]
Hexaethylene glycol ditosylate[4]

which were synthesised according to literature procedures. Solvents were laboratory reagent grade. Petroleum ether (PE) refers to the fraction of petrol boiling in the range 40 – 60 °C, dichloromethane (DCM), ethylenediaminetetraacetate (EDTA), tetrahydrofuran (THF), dimethyl sulfoxide (DMSO), dimethylformamide (DMF). $^{1}H$ and $^{13}C$ NMR spectra were recorded on either a Bruker Avance 400 MHz or a Bruker Avance 600 MHz spectrometer. Chemical shifts are reported in parts per million and referenced to residual solvent peaks ($CDCl_3$: $^{1}H$ δ 7.26 ppm, $^{13}C$ δ 77.16 ppm; [D$_6$]DMSO: $^{1}H$ δ 2.50 ppm; $^{13}C$ δ 39.52 ppm). Coupling constants (*J*) are reported in Hertz (Hz). Standard abbreviations indicating multiplicity were used as follows: m = multiplet, q = quartet, quin = quintet, t = triplet, dt = double triplet, d = doublet, dd = double doublet, s = singlet, br = broad. Electrospray mass spectra (HR ESI-MS) were collected on a Bruker micrOTOF-Q spectrometer or a Waters Synapt G2-S1 HDMS spectrometer.

**CAUTION: WHILE NO PROBLEMS WERE ENCOUNTERED DURING THIS WORK, AZIDES ARE EXPLOSIVE AND CARE SHOULD BE TAKEN WHEN DEALING WITH THEM.**

## 1.2. Precursors

### 1.2.1. 1

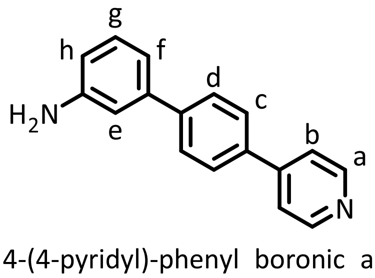


4-(4-pyridyl)-phenyl boronic acid (2.00 g, 10.0 mmol), sodium carbonate (7.99 g, 75.4 mmol), [$Pd_2(dba)_3$] (0.460 g, 0.502 mmol) and [$HP(t\text{-}bu)_3$]($BF_4$) (0.583 g, 2.01 mmol) were combined in degassed DMF/water (3:1 v/v, 50 mL). 3-iodoaniline (2.64 g, 12.1 mmol) was added neat and the mixture was heated to 100 °C with stirring under inert atmosphere overnight. After cooling, the mixture was diluted with DCM (100 mL) and water (100 mL). The aqueous phase was extracted with $CHCl_3$/2-propanol (3:1 v/v, 3 x 50 mL). The combined organic layers were washed with water (3 x 100 mL) and brine (50 mL), dried over $Na_2SO_4$, filtered and the solvent removed under reduced pressure. Column chromatography (eluting with acetone/DCM, gradient 1:4 to 4:1) gave the product as a pale yellow solid after removal of solvent. Yield: 1.15 g (46%). $^{1}H$ NMR (400 MHz, $CDCl_3$, 298 K) *δ*: 8.64 (d, *J* = 6.2 Hz, 2H, $H_a$), 7.89 (d, *J* = 8.4 Hz, 2H, $H_c$), 7.75 (d, *J* =6.2 Hz, 2H, $H_b$), 7.70 (d, *J* = 8.4 Hz, 2H, $H_d$), 7.13 (t, *J* = 7.8 Hz, 1H, $H_g$), 6.91 (t, *J* = 2.0 Hz, 1H, $H_e$), 6.84 (dt, *J* = 7.8, 1.2 Hz, 1H, $H_f$), 6.59 (ddd, *J* = 8.0, 2.3, 0.9 Hz, 1H, $H_h$), 5.20 (s, 2H, $NH_2$); $^{13}C$ NMR (100 MHz, [D$_6$]DMSO, 298 K) *δ*: 150.7, 149.7, 147.0, 142.3,

140.4, 136.1, 130.0, 127.7, 127.6, 121.4, 114.8, 114.0, 112.5; HR ESI-MS (DCM/MeOH) *m/z* = 247.1243 [**M** + H]$^+$ (calc. for $C_{17}H_{15}N_2$, 247.1235).

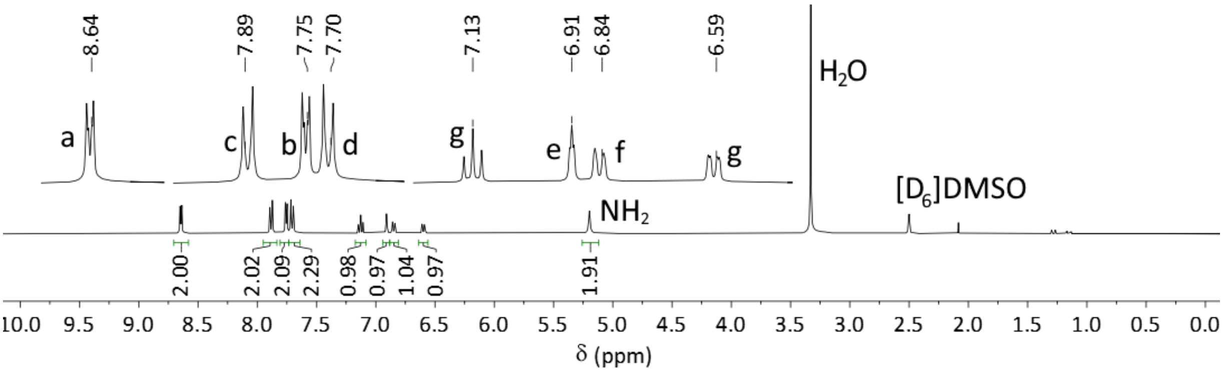


**Figure 1.1** $^1$H NMR spectrum (400 MHz, [D$_6$]DMSO, 298 K) of **1**.

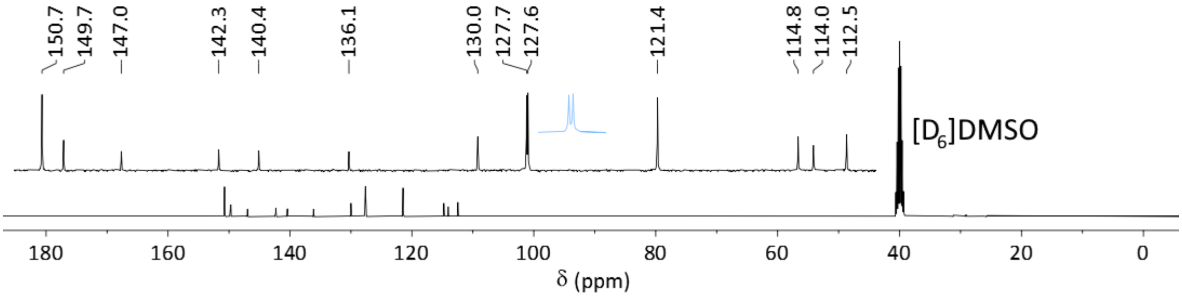


**Figure 1.2** $^{13}$C NMR spectrum (100 MHz, [D$_6$]DMSO, 298 K) of **1**.

### 1.2.2. 2

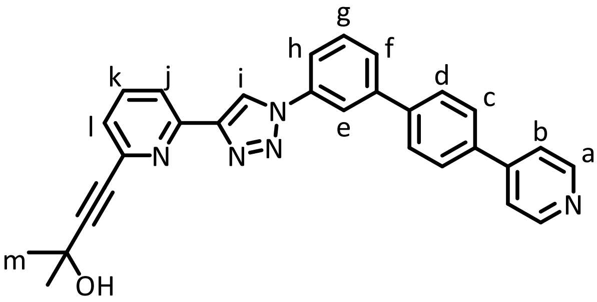


A suspension of **1** (1.30 g, 5.28 mmol) and $HCl_{aq}$ (40 mL, 6 M) was stirred in an ice bath. Sodium nitrite (0.728 g, 10.6 mmol) in water (4 mL) was added dropwise with stirring. After five minutes, sodium azide (0.686 g, 10.6 mmol) in water (4 mL) was added dropwise with stirring. After one hour, the mixture was neutralised with $NaHCO_3$ and the aqueous phase was extracted with ethyl acetate (3 x 40 mL). The combined organic layers were washed with water (50 mL) and the solvent removed under vacuum. The residue was dissolved in 4:1 DMF/water (25 mL) and to this was added 2-(2-methyl-2'-hydroxy-1-butynyl)-6-(2-(trimethylsilyl)ethynyl)-pyridine[1] (1.77 g, 6.86 mmol), sodium carbonate (1.45 g, 13.7 mmol), $CuSO_4{\cdot}5H_2O$ (0.527 g, 2.11 mmol) and sodium ascorbate (0.836 g, 4.22 mmol). After stirring at room temperature overnight, DCM (100 mL) and EDTA/$NH_4OH_{aq}$ (0.1 M, 100 mL) were added and the mixture was stirred vigorously for one hour. The layers were separated and the aqueous phase was extracted with DCM (2 x 40 mL). The combined organic layers were washed with water (5 x 100 mL) then with brine (50 mL) and the solvent removed under vacuum. Column chromatography (eluting with acetone/DCM, gradient 1:4 v/v to neat acetone) gave the product as a pale brown solid. Yield: 1.92 g (80%). $^1$H NMR (400 MHz, [D$_6$]DMSO, 298 K) δ: 9.58 (s, 1H, $H_i$), 8.67 (s,

2H, $H_a$), 8.43 (t, *J* = 2.0 Hz, 1H, $H_e$), 8.13 – 8.08 (m, 2H, $H_h$ and $H_k$), 8.03 (d, *J* = 6.5 Hz, 2H, $H_c$), 7.98 – 7.94 (m, 3H, $H_d$ and $H_j$), 7.91 (dt, *J* = 7.9, 1.2 Hz, 1H, $H_f$), 7.81 (d, *J* = 5.0 Hz, 2H, $H_b$), 7.73 (t, *J* = 8.0 Hz, 1H, $H_g$), 7.47 (dd, *J* = 7.7, 1.1 Hz, 1H, $H_l$), 5.63 (s, 1H, $H_n$), 1.51 (s, 6H, $H_m$); $^{13}$C NMR (100 MHz, [D$_6$]DMSO, 298 K) δ: 150.3, 150.0, 147.6, 146.3, 142.6, 140.9, 139.5, 137.9, 137.2, 136.8, 130.6, 127.8, 127.4, 126.9, 126.5, 121.8, 121.2, 119.5, 119.3, 118.2, 95.8 80.2, 63.6, 31.4; HR ESI-MS (DCM/MeOH) *m/z* = 458.1981 [**M** + H]$^+$ (calc. for $C_{29}H_{23}N_5O$, 458.1981). IR *v* (cm$^{-1}$) 3203, 2972, 2922, 2853, 2228, 1596, 1562, 1171, 1032, 793, 780.

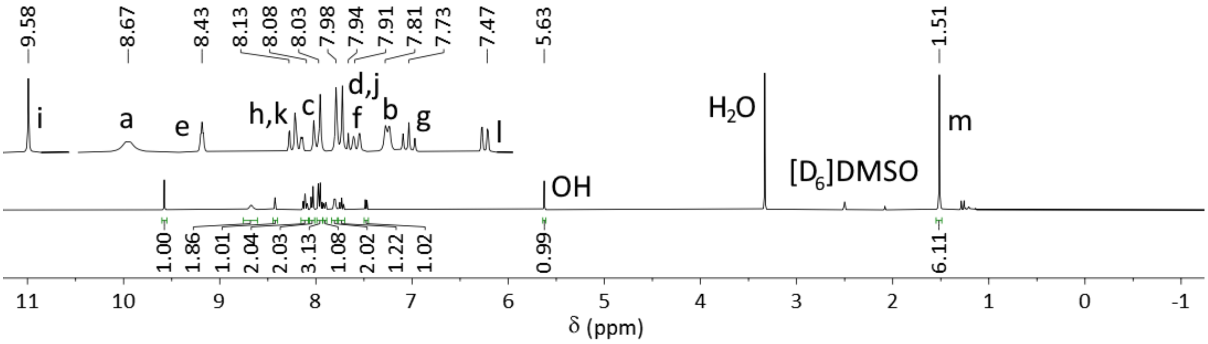


**Figure 1.3:** $^1$H NMR spectrum (400 MHz, [D$_6$]DMSO, 298 K) of **2**.

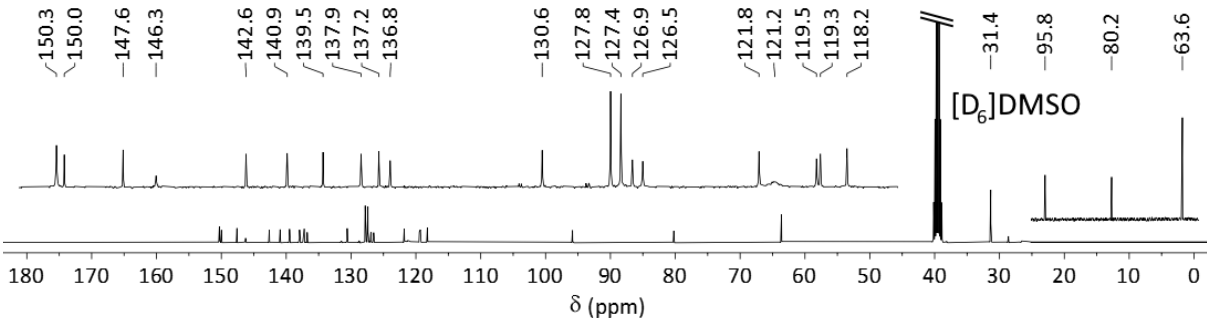


**Figure 1.4:** $^{13}$C NMR spectrum (100 MHz, [D$_6$]DMSO, 298 K) of **2**.

## 1.3. Ligands

### 1.3.1. L-mono

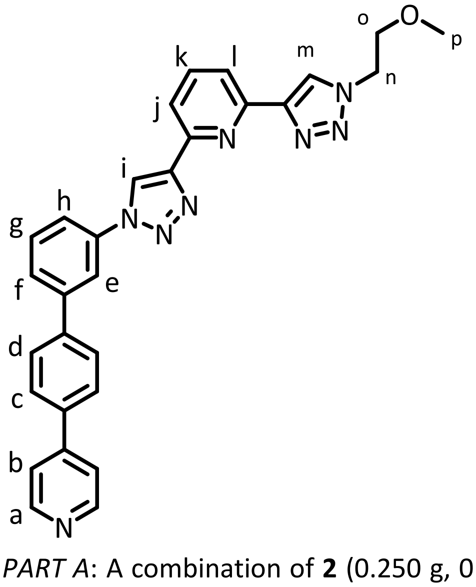


*PART A*: A combination of **2** (0.250 g, 0.546 mmol) and sodium hydroxide (0.0546 g, 1.37 mmol) in toluene (10 mL) was refluxed for 80 minutes. The solution was filtered through Celite, rinsing with

DCM and the solvent was removed under vacuum. The residue was used in *PART B* without further purification.

*PART B*: Ethylene glycol monomethyl tosylate [2] (0.151 g, 0.655 mmol) and sodium azide (0.0461 g, 0.710 mmol) were combined in 4:1 DMF/water (3 mL) and were heated at 110 °C for one hour with stirring. The resulting mixture was added to the residue from *Part A* along with $CuSO_4 \cdot 5H_2O$ (0.0545 g, 0.218 mmol) and sodium ascorbate (0.0865 g, 0.437 mmol) and additional DMF/water (4:1 v/v, 3 mL). The mixture was heated at 50 °C overnight. DCM (100 mL) and EDTA/$NH_4OH$ (0.1 M, 100 mL) were added and the mixture was stirred vigorously for one hour. The layers were separated and the aqueous layer was extracted with DCM (2 x 50 mL). The combined organic layers were washed with water (50 mL), dried over $Na_2SO_4$, filtered and the solvents were removed under reduced pressure. The residue was purified by column chromatography (eluting with DCM/acetone, gradient neat DCM to DCM/acetone 2:1) to give the desired product as an off-white solid after removal of solvent. Yield: 0.207 g (75%). $^1$H NMR (400 MHz, [D$_6$]DMSO, 298 K) δ: 9.52 (s, 1H, $H_i$), 8.69 – 8.67 (m, 3H, $H_a$, and $H_m$), 8.39 (t, *J* = 2.0 Hz, 1H, $H_e$), 8.09 – 7.98 (m, 8H, $H_c$, $H_d$, $H_h$, $H_j$, $H_k$, and $H_l$), 7.93 (d, *J* = 8.2 Hz, 1H, $H_f$), 7.81 (d, *J* = 6.2 Hz, 2H, $H_b$), 7.77 (t, *J* = 7.9 Hz, 1H, $H_g$), 4.64 (t, *J* = 5.1 Hz, 2H, $H_n$), 3.81 (t, *J* = 5.1 Hz, 2H, $H_o$), 3.28 (s, 3H, $H_p$). $^1$H DOSY NMR *D* ($\times10^{-10}$ m$^2$ s$^{-1}$): 1.42. $^{13}$C NMR (100 MHz, [D$_6$]DMSO) δ: 150.3, 150.1, 149.4, 148.1, 147.0, 146.3, 141.1, 139.6, 138.4, 137.3, 136.8, 130.7, 127.8, 127.5, 127.1, 124.1, 121.9, 121.2, 119.7, 118.8, 118.7, 118.4, 70.2, 58.1, 49.7. HR ESI-MS (DCM/MeOH) *m/z* = 458.1981 [**M** + H]$^+$ (calc. for $C_{29}H_{23}N_5O$, 458.1981). IR *v* (cm$^{-1}$) 3134, 2924, 2861, 1594, 1455, 1394, 1209, 1108, 940, 810.

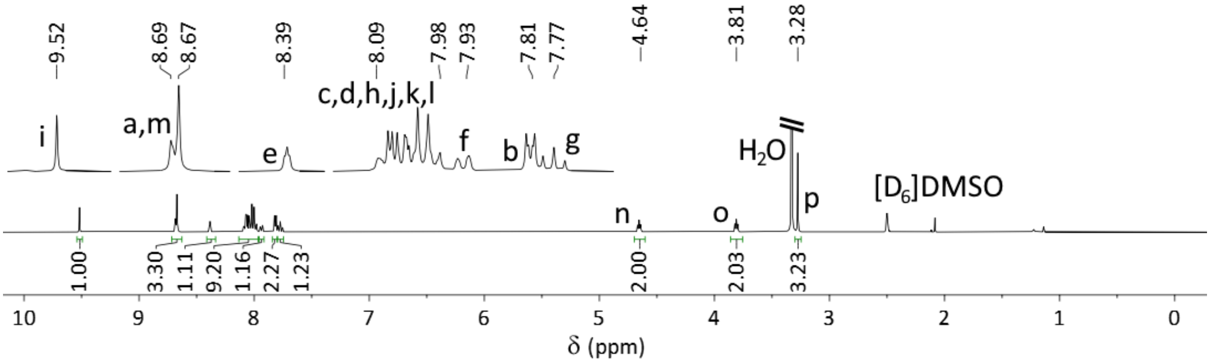


**Figure 1.5** $^1$H NMR spectrum (400 MHz, [D$_6$]DMSO, 298 K) of **L-mono**.

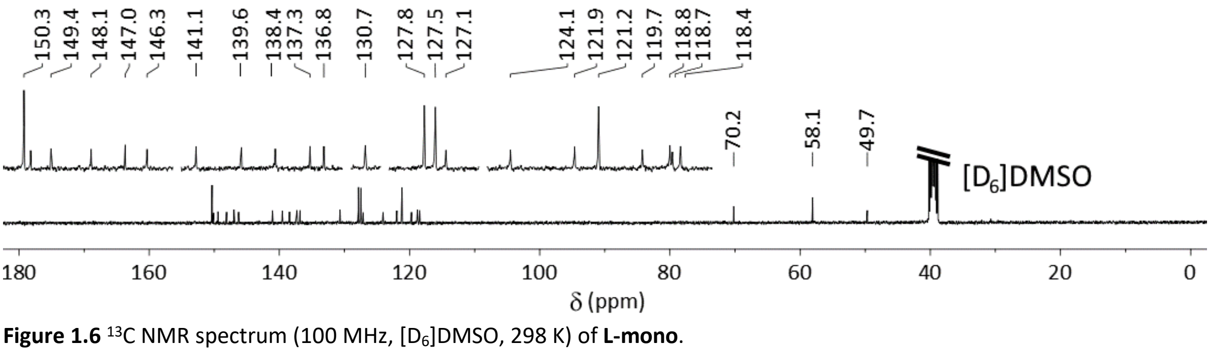


**Figure 1.6** $^{13}$C NMR spectrum (100 MHz, [D$_6$]DMSO, 298 K) of **L-mono**.

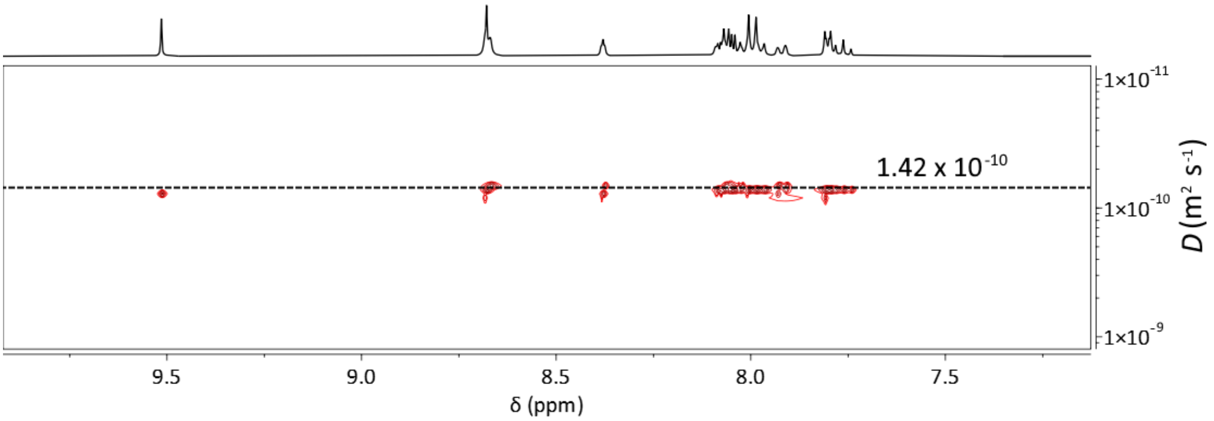


**Figure 1.7** Partial $^1$H DOSY NMR spectrum (400 MHz, [D$_6$]DMSO, 298 K) of **L-mono**.

### 1.3.2. L-4PEG

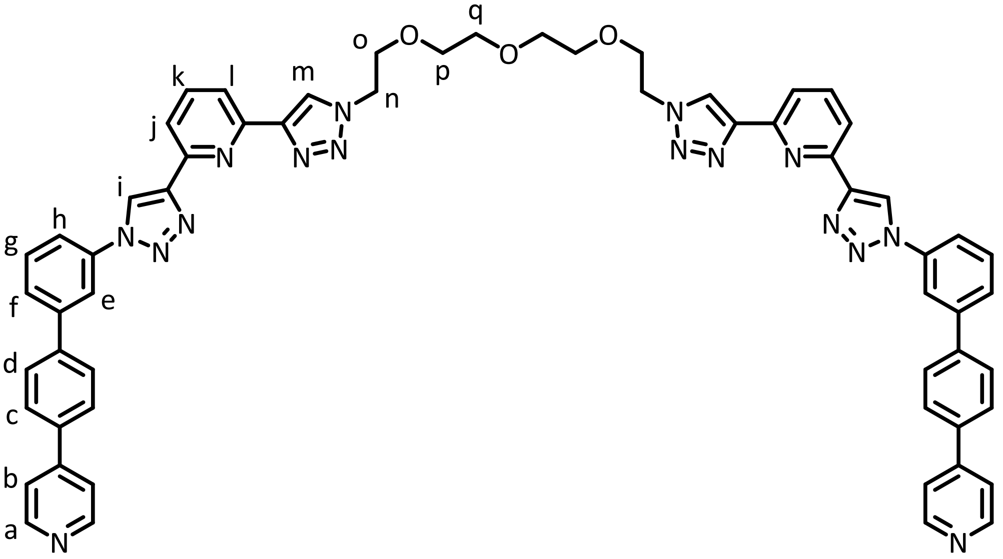

*PART A*: A combination of **2** (0.145 g, 0.317 mmol) and sodium hydroxide (0.032 g, 0.79 mmol) in toluene (8 mL) was refluxed for 80 minutes. The solution was filtered through cotton wool, and the solvent was removed under vacuum. The residue was passed through a silica plug (eluting with acetone) and the solvent removed under vacuum. The mass of the residue was 0.089 g.

*PART B*: Tetraethylene glycol ditosylate[3] (0.053 g, 0.11 mmol) and sodium azide (0.014 g, 0.22 mmol) were combined in DMF (1 mL) and were heated at 110 °C for one hour with stirring. The resulting mixture was added to the residue from Part A along with $CuSO_4·5H_2O$ (0.013 g, 0.053 mmol) and sodium ascorbate (0.021 g, 0.11 mmol) and DMF (6 mL) and water (1 mL). The mixture was heated at 50 °C overnight under $N_2$. DCM (100 mL) and EDTA/$NH_4OH$ (0.1 M, 100 mL) were added and the mixture was stirred vigorously for one hour. The organic phase was separated, and the solvent removed under vacuum. The residue was taken up in DCM (100 mL) and washed with water (2 x 100 mL) and the solvent removed under vacuum to give the product as a white solid. Yield: 100 mg (90%). $^1$H NMR (400 MHz, [D$_6$]DMSO, 298 K) δ: 9.42 (s, 2H, $H_i$), 8.65 (d, $J$ = 6.3 Hz, 4H, $H_a$) 8.59 (s, 2H, $H_m$), 8.31 (t, $J$ = 2.0 Hz, 2H, $H_e$), 8.03 – 7.92 (m, 16H, $H_c$, $H_d$, $H_h$, $H_j$, $H_k$, $H_l$), 7.87 (d, $J$ = 7.9 Hz, 2H, $H_f$), 7.76 (d, $J$ = 6.2 Hz, 4H, $H_b$), 7.71 (t, $J$ = 7.9 Hz, 2H, $H_g$), 4.54 (t, $J$ = 5.5 Hz, 4H, $H_n$), 3.78 (t, $J$ = 4.8 Hz, 4H, $H_o$), 3.44 – 3.43 (m, 8H, $H_p$, $H_q$). $^1$H DOSY NMR $D$ (x10$^{-10}$ m$^2$ s$^{-1}$): 1.15. $^{13}$C NMR (100 MHz, [D$_6$]DMSO) δ: 150.7, 150.5, 149.8, 148.5, 147.4, 146.7, 141.4, 140.0, 138.8, 137.7, 137.2, 131.0, 128.2, 127.9, 127.5, 124.4, 122.2, 121.6, 120.0, 119.2, 119.1, 118.7, 71.0, 70.0, 69.1, 50.2. HR ESI-MS (MeOH) $m/z$ = 1065.4379

[**M** + Na]$^+$ (calc. for $C_{60}H_{50}N_{16}NaO_3$, 1065.4149). IR *v* (cm$^{-1}$) 3139, 3076, 2928, 2857, 1593, 1570, 1485, 1421, 1095, 1029, 811, 790.

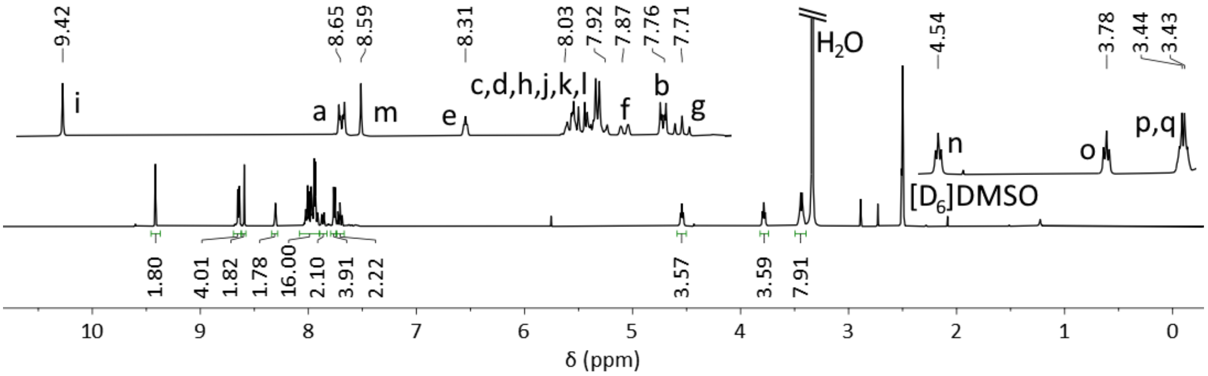


**Figure 1.8** $^{1}$H NMR spectrum (400 MHz, [D$_6$]DMSO, 298 K) of **L-4PEG**.

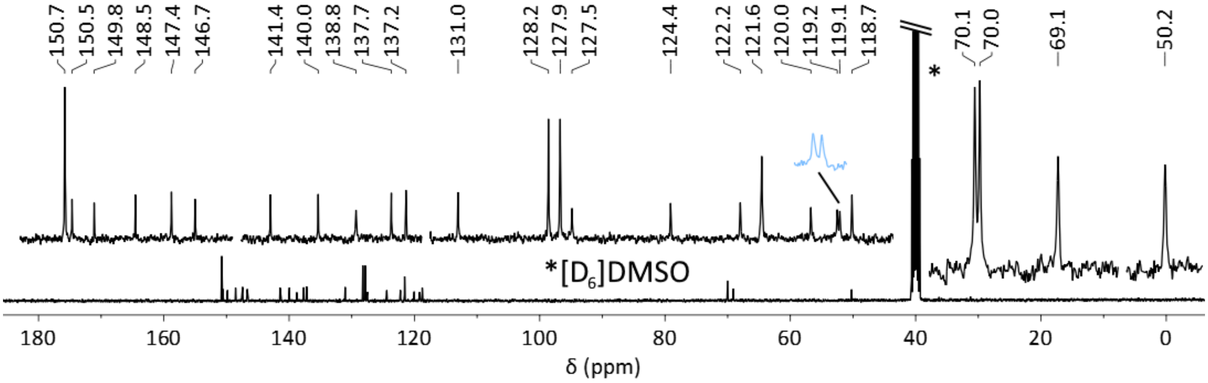


**Figure 1.9** $^{13}$C NMR spectrum (100 MHz, [D$_6$]DMSO, 298 K) of **L-4PEG**.

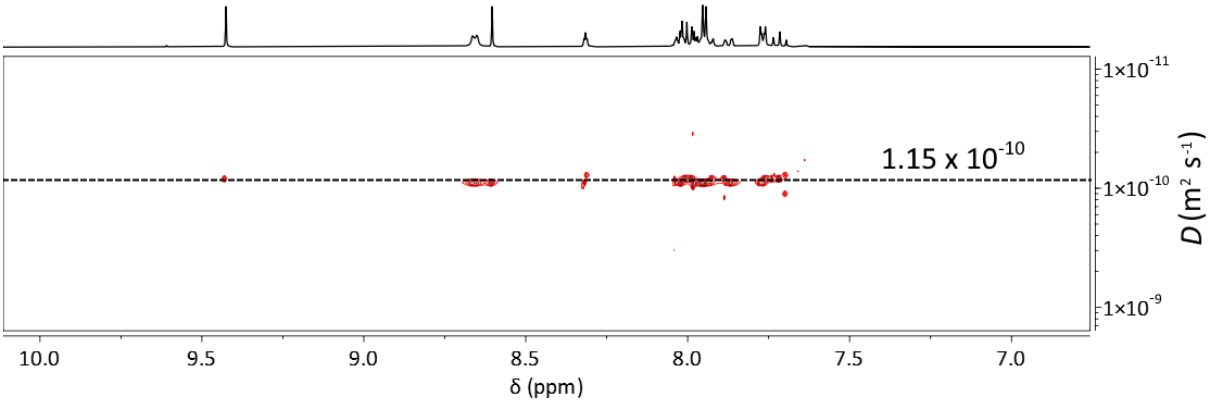


**Figure 1.10** Partial $^{1}$H DOSY NMR spectrum (400 MHz, [D$_6$]DMSO, 298 K) of **L-4PEG**.

## 1.4. L-6PEG

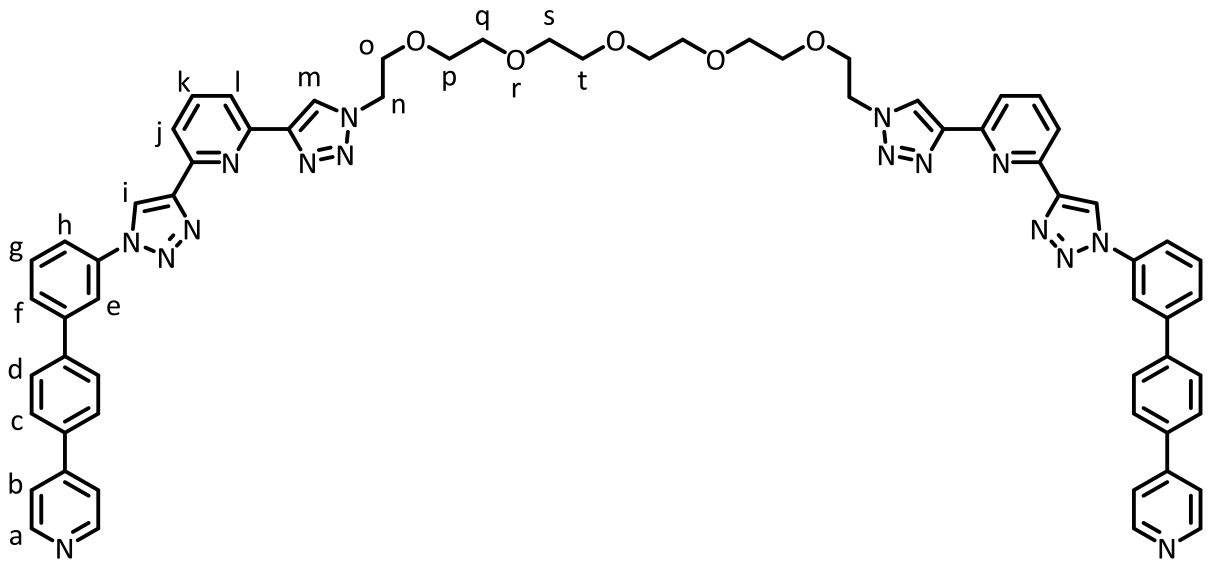

*PART A*: A combination of **2** (0.250 g, 0.546 mmol) and sodium hydroxide (0.055 g, 1.4 mmol) in toluene (10 mL) was refluxed for 80 minutes. The solution was filtered through cotton wool, and the solvent was removed under vacuum. The residue was passed through a silica plug (eluting with acetone) and the solvent removed under vacuum. The mass of the residue was 0.160 g.

*PART B*: Hexaethylene glycol ditosylate[4] (0.113 g, 0.191 mmol) and sodium azide (0.026 g, 0.40 mmol) were combined in DMF (1 mL) and were heated at 110 °C for one hour with stirring. The resulting mixture was added to the residue from PART A along with $CuSO_4{\cdot}5H_2O$ (0.024 g, 0.095 mmol) and sodium ascorbate (0.038 g, 0.19 mmol) and DMF (6 mL) and water (1 mL). The mixture was heated at 50 °C overnight under $N_2$. DCM (100 mL) and EDTA/$NH_4OH$ (0.1 M, 100 mL) were added and the mixture was stirred vigorously for one hour. The organic phase was separated, and the solvent removed under vacuum. The residue was taken up in DCM (100 mL) and washed with water (2 x 100 mL) and the solvent removed under vacuum to give the product as a white solid. Yield: 211 mg (98%). $^1$H NMR (400 MHz, [D$_6$]DMSO, 298 K) δ: 9.45 (s, 2H, $H_i$), 8.66 – 8.64 (m, 6H, $H_a$, $H_m$), 8.34 (t, *J* = 1.9 Hz, 2H, $H_e$), 8.06 – 7.93 (m, 16H, $H_c$, $H_d$, $H_h$, $H_j$, $H_k$, $H_l$), 7.88 (d, *J* = 7.8 Hz, 2H, $H_f$), 7.77 (d, *J* = 6.4 Hz, 4H, $H_b$), 7.72 (t, *J* = 8.0 Hz, 2H, $H_g$), 4.60 (t, *J* = 5.5 Hz, 4H, $H_n$), 3.84 (t, *J* = 4.7 Hz, 4H, $H_o$), 3.48 – 3.46 (m, 4H, $H_p$), 3.39 - 3.37 (m, 4H, $H_q$), 3.31 – 3.29 (m, 4H, $H_r$), 3.26 – 3.24 (m, 4H, $H_s$). $^1$H DOSY NMR *D* (x$10^{-10}$ m$^2$ s$^{-1}$): 1.12. $^{13}$C NMR (100 MHz, [D$_6$]DMSO) δ: 150.3, 150.1, 149.4, 148.1, 147.0, 146.2, 141.0, 139.5, 138.4, 137.2, 136.8, 131.4, 130.6, 127.8, 127.4, 127.1, 124.0, 121.8, 121.1, 119.6, 118.7, 118.3, 69.6 (three coincident peaks), 69.5, 68.6, 49.8. HR ESI-MS (MeOH) *m/z* = 1153.4924 [**M** + Na]$^+$ (calc. for $C_{64}H_{58}N_{16}NaO_5$, 1153.4674). IR *v* (cm$^{-1}$) 3144, 3073, 2931, 2860, 1592, 1485, 1093, 1028, 812, 794.

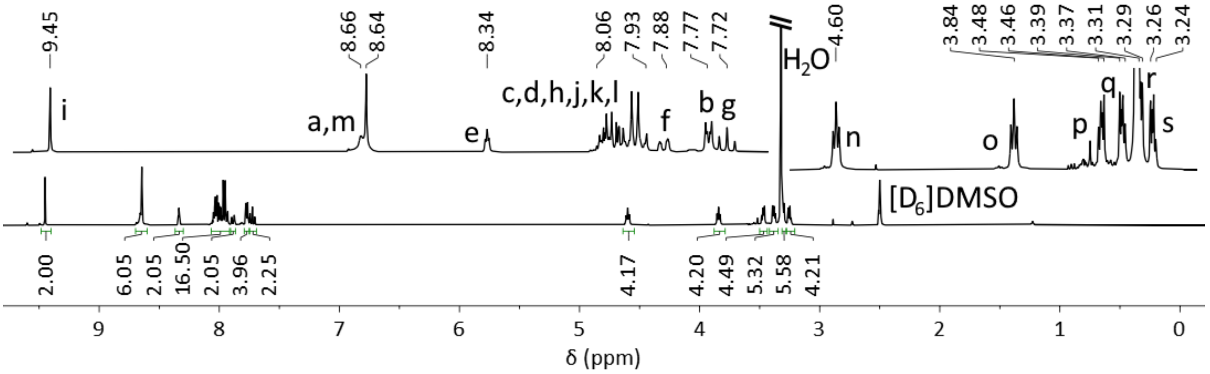


**Figure 1.11** $^1$H NMR spectrum (400 MHz, [D$_6$]DMSO, 298 K) of **L-6PEG**.

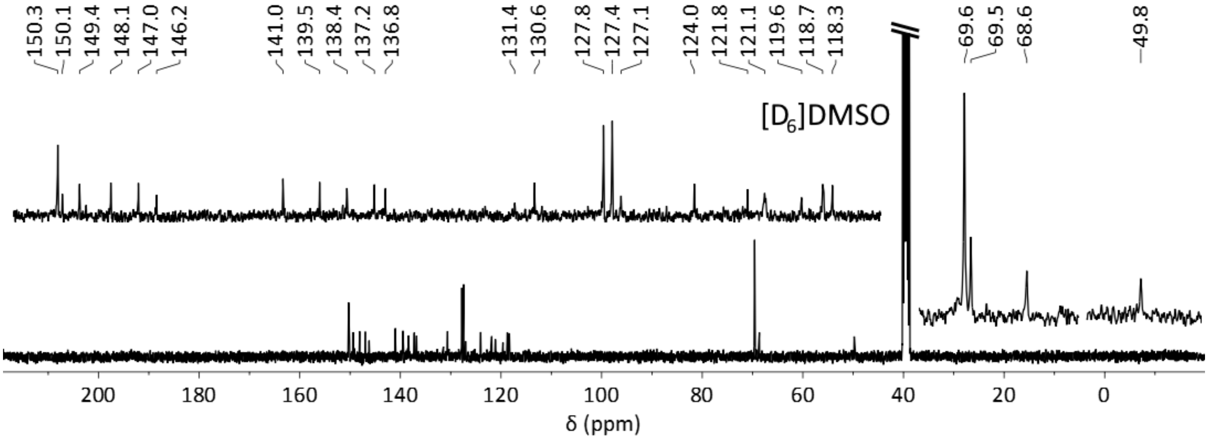


**Figure 1.12** $^{13}C$ NMR spectrum (100 MHz, [D$_6$]DMSO, 298 K) of **L-6PEG**.

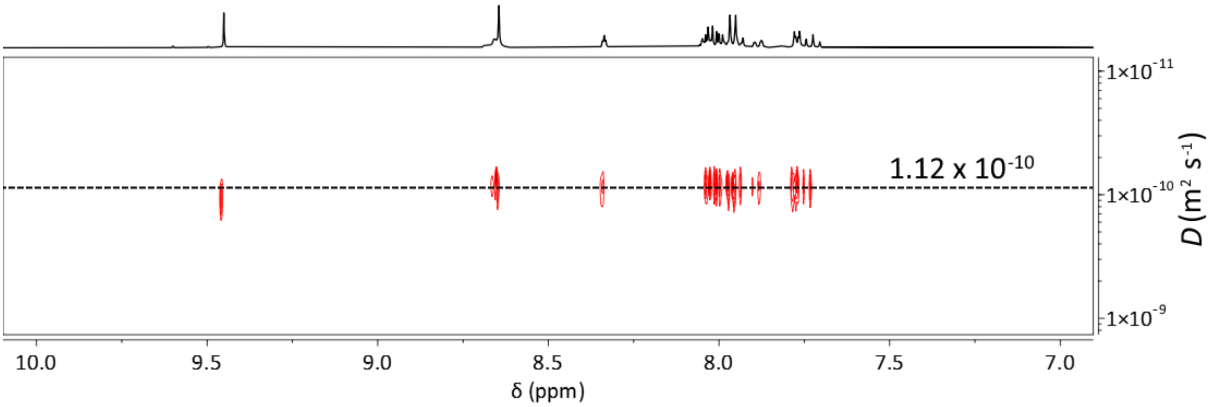


**Figure 1.13** Partial $^{1}H$ DOSY NMR spectrum (400 MHz, [D$_6$]DMSO, 298 K) of **L-6PEG**.

## 1.5. Complexes

### 1.5.1. CYCLE-mono

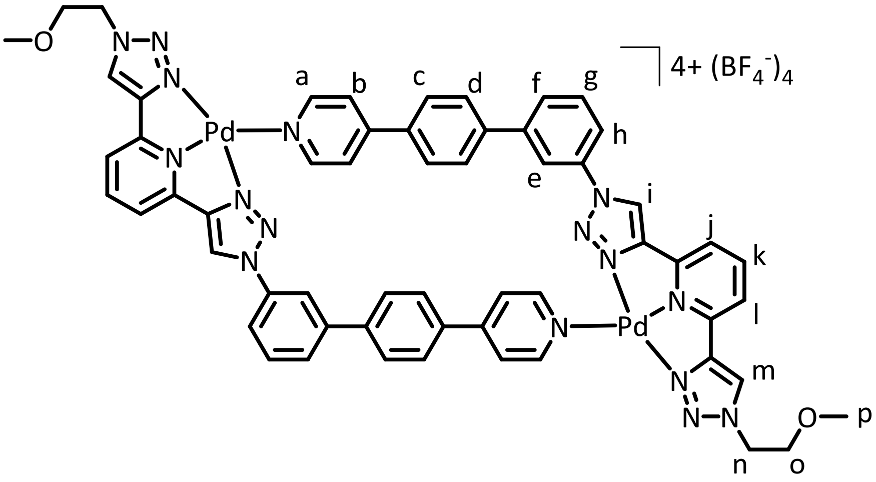


The combination of **L-mono** (3.78 mg, 7.50 µmol) and $[Pd(CH_3CN)_4](BF_4)_2$ (3.35 mg, 7.50 µmol) from stock solutions in [D$_6$]DMSO (500 µL) for a concentration of each component of 15 mM gave quantitative solution phase conversion into the product, [Pd$_2$(**L-mono**)$_2$](BF$_4$)$_4$ (**CYCLE-mono**). Addition of acetonitrile and vapour diffusion of diethyl ether gave the product as a white powder. Yield 3.89 mg (66%). $^{1}H$ NMR (400 MHz, [D$_6$]DMSO, 298 K) δ: *integration given per ligand* 10.30 (s, 1H, H$_i$), 9.49

(s, 1H, $H_m$), 9.13 (d, $J$ = 5.2 Hz, 2H, $H_a$), 8.73 (t, $J$ = 7.9 Hz, 1H, $H_k$), 8.48 – 8.47 (m, 2H, $H_e$, $H_j$), 8.43 (d, $J$ = 7.0 Hz, 2H, $H_b$), 8.30 (d, $J$ = 8.9 Hz, 1H, $H_l$), 8.23 – 8.10 (m, 6H, $H_c$, $H_d$, $H_f$, $H_h$), 7.95 (t, $J$ = 8.0 Hz, $H_g$), 4.94 (t, $J$ = 4.4 Hz, 2H, $H_n$), 3.88 (t, $J$ = 4.1 Hz, 2H, $H_o$), 3.34 (s, 3H, $H_p$). $^1$H DOSY NMR $D$ (x10$^{-10}$ m$^2$ s$^{-1}$): 0.83. HR ESI-MS (DMSO/acetonitrile) $m/z$ = 303.5668 [**CYCLE-mono**]$^{4+}$ (calc. for [$Pd_2(C_{29}H_{24}N_8O)_2$], 303.5560); 404.4157 [**CYCLE-mono** – H]$^{3+}$ (calc. for [$Pd_2(C_{29}H_{24}N_8O)(C_{29}H_{23}N_8O$], 404.4158); 411.0814 [**CYCLE-mono** + F$^-$]$^{3+}$ (calc. for [$Pd_2(C_{29}H_{24}N_8O)_2$]F, 411.0845; 694.1281 [**CYCLE-mono** + 2BF$_4^-$]$^{2+}$ (calc. for [$Pd_2(C_{29}H_{24}N_8O)_2$]($BF_4$)$_2$, 694.1165). IR $v$ (cm$^{-1}$) 3124, 1618, 1591, 1478, 1290, 1040, 817, 804.

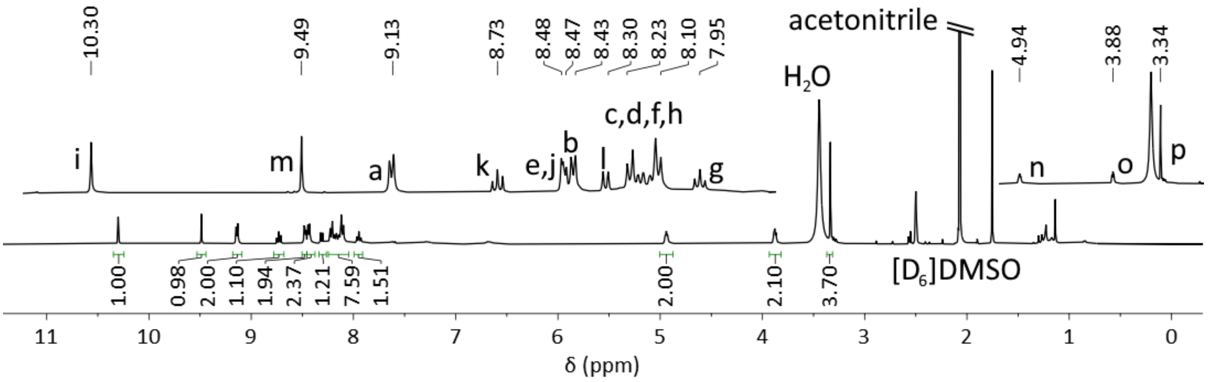


**Figure 1.14** $^1$H NMR spectrum (400 MHz, [D$_6$]DMSO, 298 K) of **CYCLE-mono**.

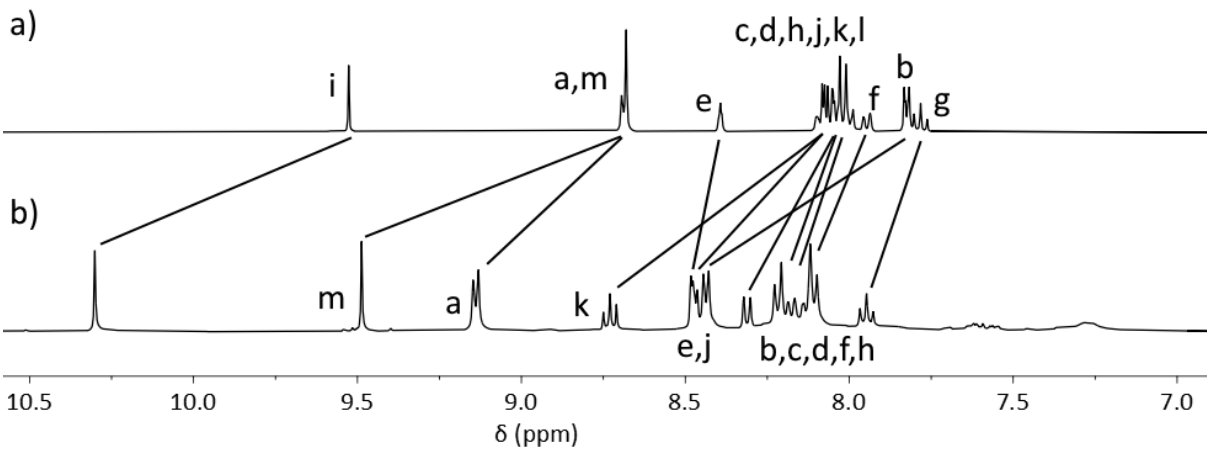


**Figure 1.15** Partial stacked $^1$H NMR spectra (400 MHz, [D$_6$]DMSO, 298 K) of a) **L-mono** and b) **CYCLE-mono**.

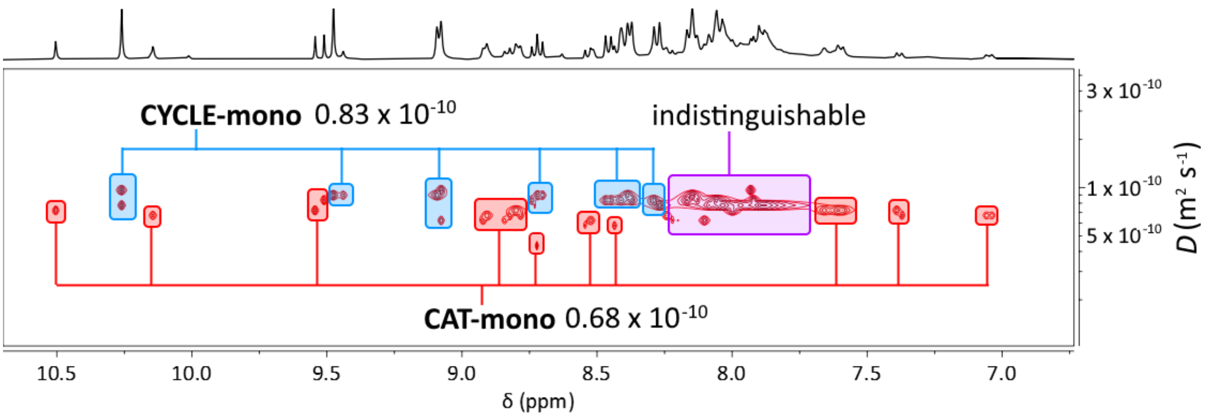


**Figure 1.16** Partial $^1$H DOSY NMR spectrum (400 MHz, [D$_6$]DMSO, 298 K) of the 1:1 combination of **L-mono** and Pd(II) (both at 60 mM). Some diffusional peaks were clearly identifiable as pertaining to **CYCLE-mono**, some to **CAT-mono**, while other overlapping peaks were indistinguishable.

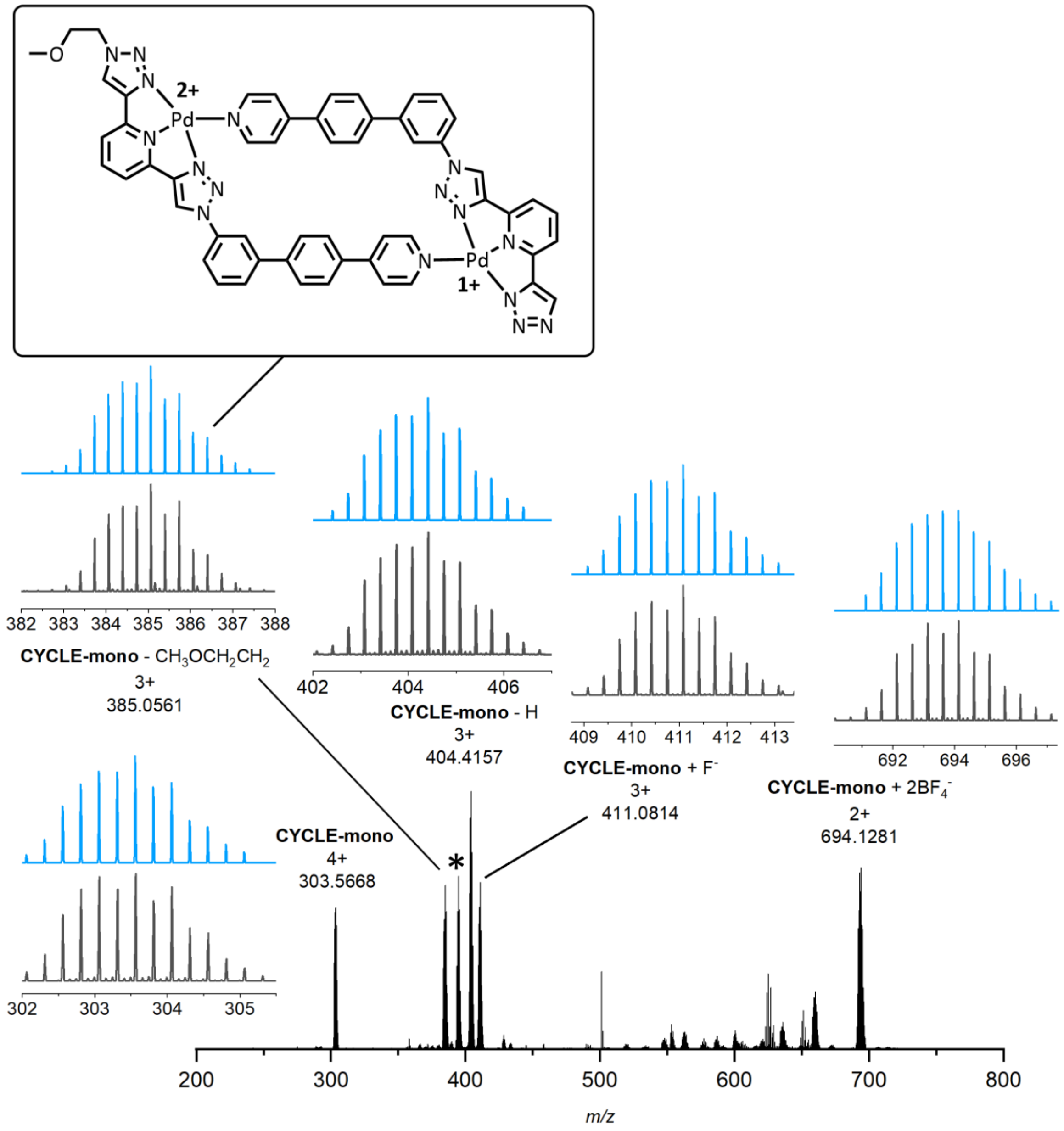


**Figure 1.17** Mass spectrum (DMSO/DMF) of **CYCLE-mono**. Observed in black, calculated in blue. Note the peak at 385.0561 *m/z* is derived from cleavage of the substituent of the peripheral triazole, and formation of the associated triazolato species.[5] There is also a peak at 395.0658 (denoted with an asterisk, *) which arises from fragmentation of the peak at 404.4157, which we were unable to conclusively identify.

We utilised MS/MS to probe the origin on the 404.4157 [**CYCLE-mono** – H]$^{3+}$ peak (Figure 1.18), as well as other species.

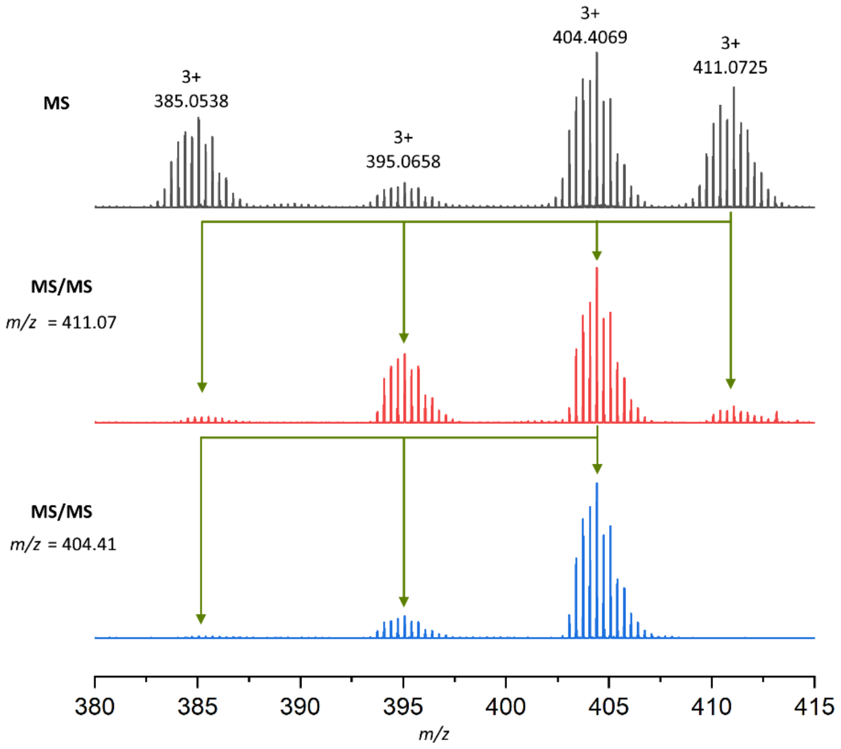

**Figure 1.18** Partial MS (MS/MS) spectra for **CYCLE-mono** (DMSO/$CH_3CN$).

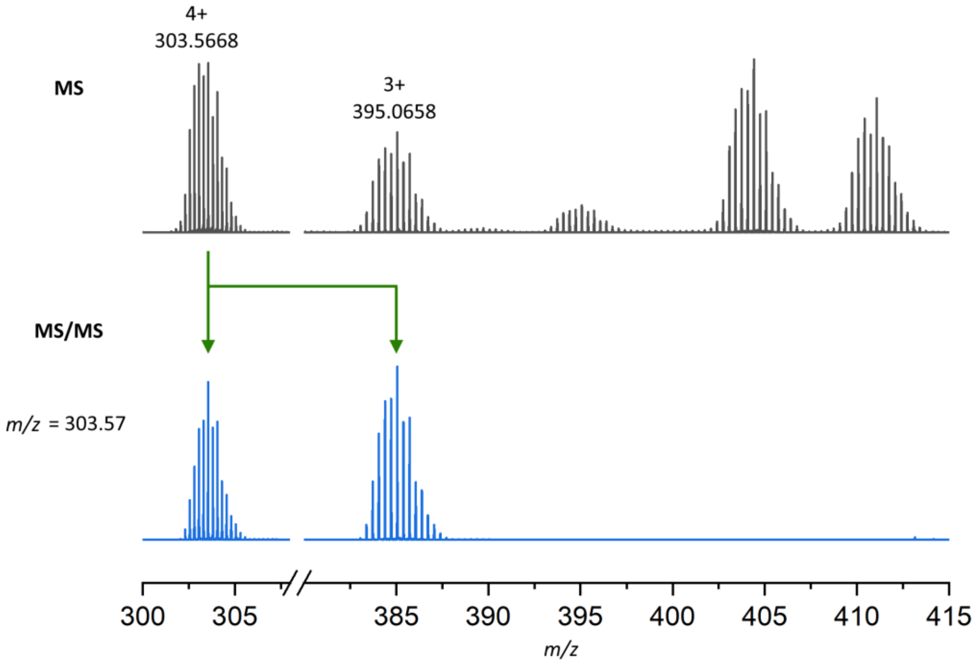

**Figure 1.19** Partial MS (MS/MS) spectra for **CYCLE-mono** (DMSO/$CH_3CN$).

From the MS/MS we were able to ascertain that the 404.4069 peak was derived from the 411.0725 peak (byproduct of formation: HF) and the 385.0538 peaks was derived from the 404.4069 peak (byproduct of formation $CH_3OCH_2CH_2$-) and possibly the 411.0725 peak. It also originates from the [**CYCLE-mono**]$^{4+}$ peak. Cleavage of substituents from triazoles for formation of triazalato species is known in the literature.[5]

**Table 1.1** Identity and origin of 3+ species. ***Note: MS/MS on the 395.0658 and 385.0538 fragments showed that neither originates from the other.***

| *m/z* | Identity | Origin | Byproduct on formation |
|---|---|---|---|
| 404.4069 | [**CYCLE-mono** – H]$^{3+}$ | [**CYCLE-mono** + F]$^{3+}$ | HF |
| 395.0658 | [unknown]$^{3+}$ | [**CYCLE-mono** – H]$^{3+}$ (and possibly [**CYCLE-mono** + F]$^{3+}$) | unknown |
| 385.0538 | [**CYCLE-mono** – $CH_3OCH_2CH_2$ | [**CYCLE-mono** – H]$^{3+}$, [**CYCLE-mono**]$^{4+}$ (and possibly [**CYCLE-mono** + F]$^{3+}$) | $CH_3OCH_2CH_2$- |

### 1.5.2. CAT-mono

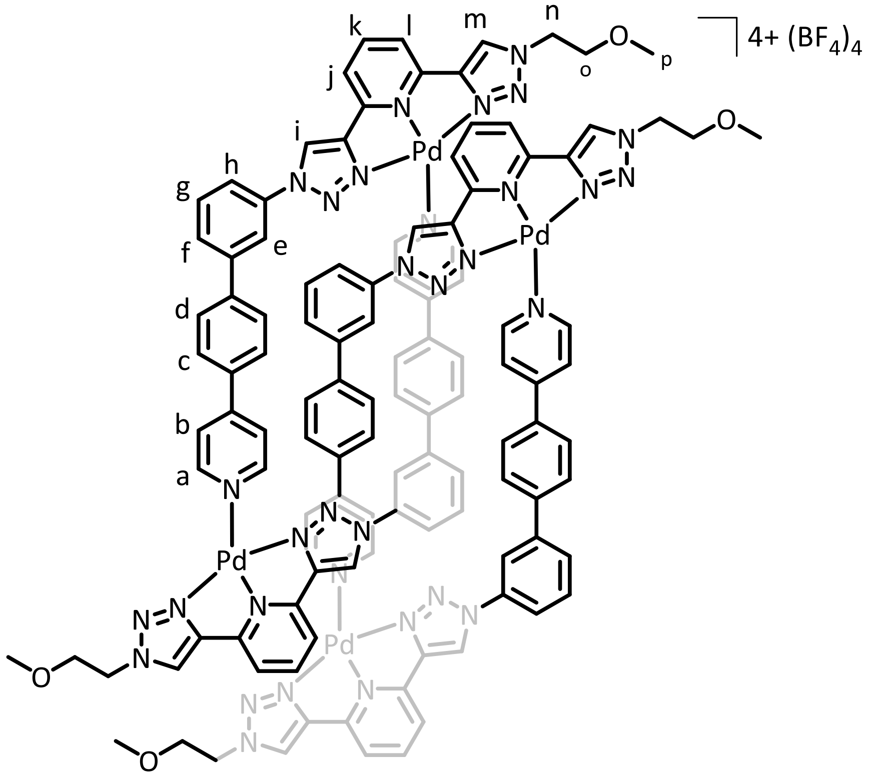


*In proton labelling for this compound, normal labels (e.g. $H_a$ or a) are the 'outer' environment, labels with a prime symbol (e.g. $H_{a'}$ or a') are the 'inner' environment.*

For the 1:1 combination of **L-mono** and $[Pd(CH_3CN)_4](BF_4)_2$ in [D$_6$]DMSO above the 15 mM concentration, a set of new peaks with two peaks per ligand environment were observed. This catenated species $[Pd_4($**L-mono**$)_4](BF_4)_8$ (**CAT-mono**) was characterised solely in solution as part of an equilibrium mixture. $^1$H DOSY NMR *D* (x10$^{-10}$ m$^2$ s$^{-1}$): 0.68. HR ESI-MS (DMSO/DMF) *m/z* = 953.4899 [**CAT-mono** + 5BF$_4^-$]$^{3+}$ (calc. for $[Pd_4(C_{29}H_{24}N_8O)_4](BF_4)_5$, 953.4899); HR ESI-MS (DMSO/acetonitrile) *m/z* = 1474.2393 [**CAT-mono** + 6BF$_4^-$]$^{2+}$ (calc. for $[Pd_4(C_{29}H_{24}N_8O)_4](BF_4)_6$, 1474.2348).

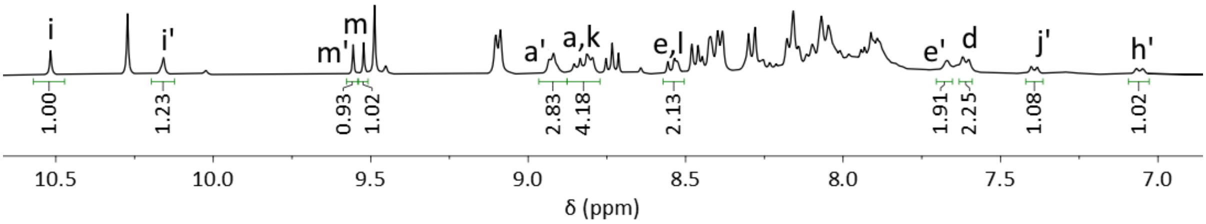


**Figure 1.20** Partial $^{1}H$ NMR spectrum (400 MHz, [$D_6$]DMSO, 298 K) of the 1:1 combination of **L-mono** and Pd(II) at 60 mM, with peaks clearly identifiable as pertaining to **CAT-mono** labelled and identified.

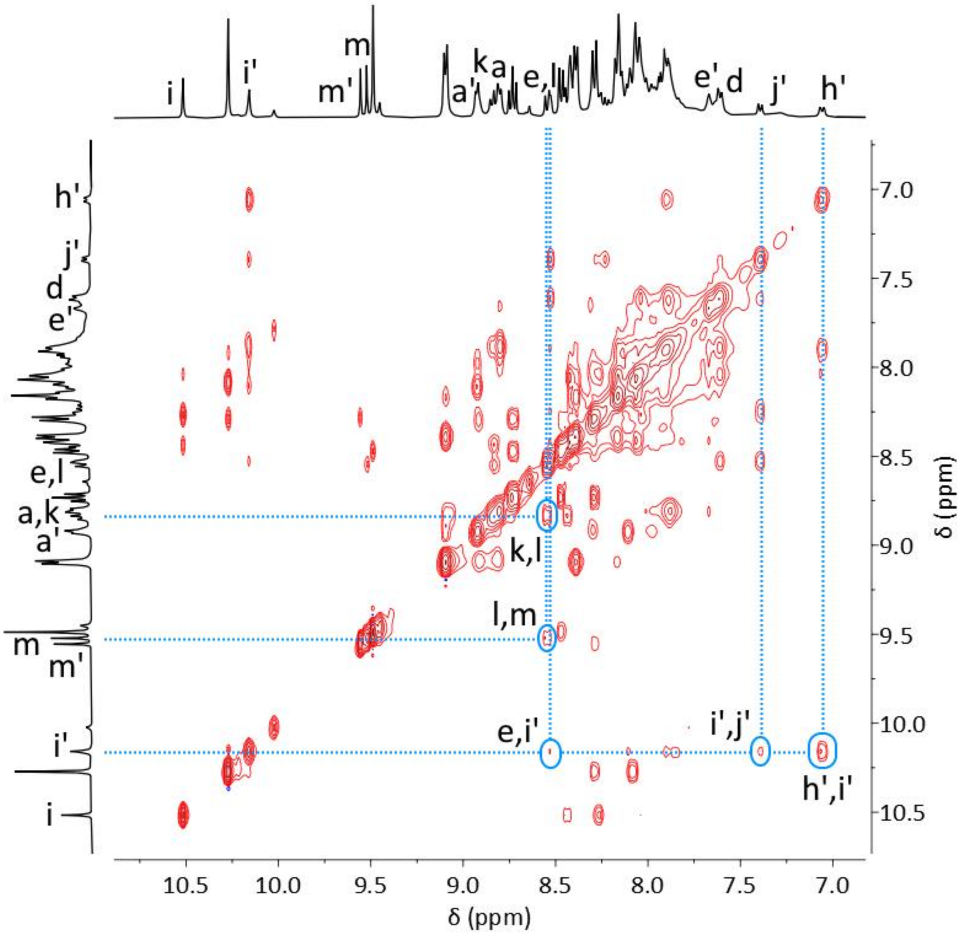


**Figure 1.21** Partial $^{1}H$ NOESY NMR spectrum (400 MHz, [$D_6$]DMSO, 298 K, 200 ms) for the 1:1 combination of **L-mono** and Pd(II) at 60 mM. Through-space couplings for **CAT-mono** highlighted.

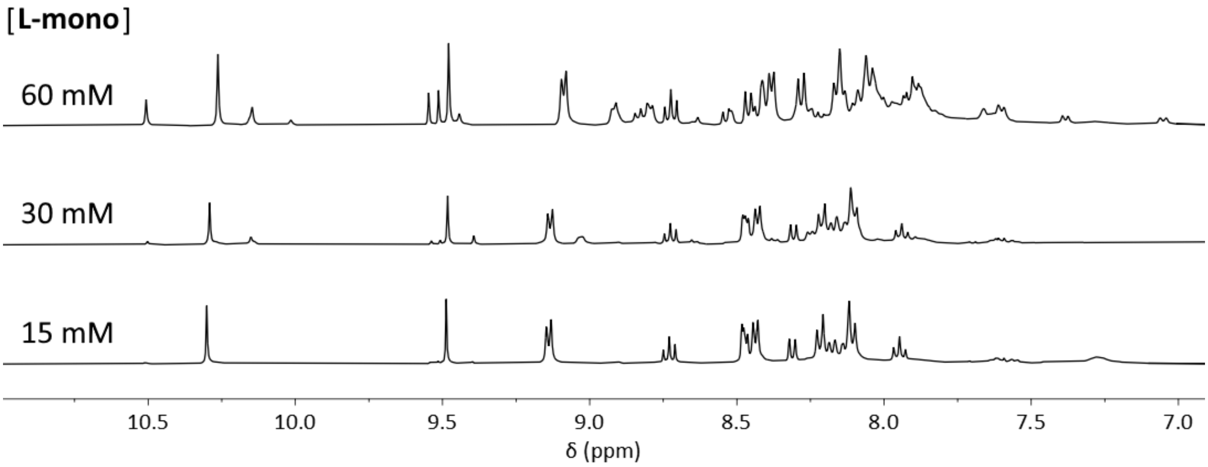


**Figure 1.22** Partial stacked spectra (400 MHz, [$D_6$]DMSO, 298 K) for the 1:1 combination of **L-mono** and Pd(II) at different concentrations. As concentration decreases, the proportion of **CAT-mono** relative to **CYCLE-mono** decreases.

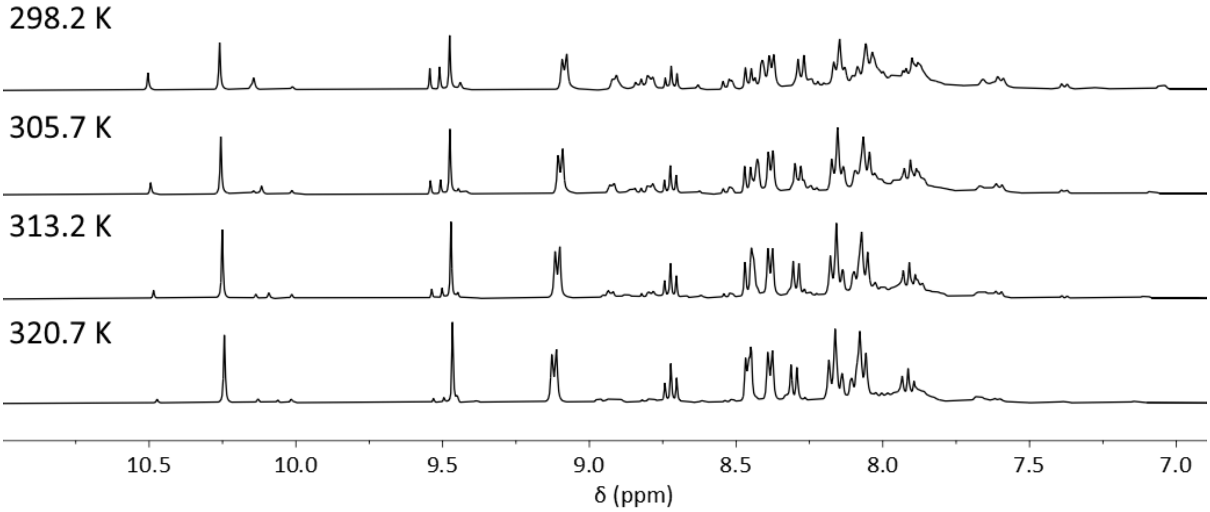


**Figure 1.23** Partial stacked spectra (400 MHz, [$D_6$]DMSO) for the 1:1 combination of **L-mono** and Pd(II) (60 mM) at different temperatures. As temperature increases, the proportion of **CAT-mono** relative to **CYCLE-mono** decreases.

Expressing the reaction as:

2 x **CYCLE-mono** $\rightleftharpoons$ **CAT-mono**

**Table 1.2** Concentrations of **CYCLE-mono** and **CAT-mono** as a function of temperature, from a starting concentration of **L-mono** of 60 mM, together with $K_{eq}$ at each temperature.

| Temperature (K) | [**CYCLE-mono**] (mM) | [**CAT-mono**] (mM) | $K_{eq}$ |
|---|---|---|---|
| 298.2 | 17.3 | 6.3 | 21.2 |
| 305.7 | 21.3 | 4.3 | 9.6 |
| 313.2 | 23.7 | 3.2 | 5.6 |
| 320.7 | 26.7 | 1.7 | 2.4 |

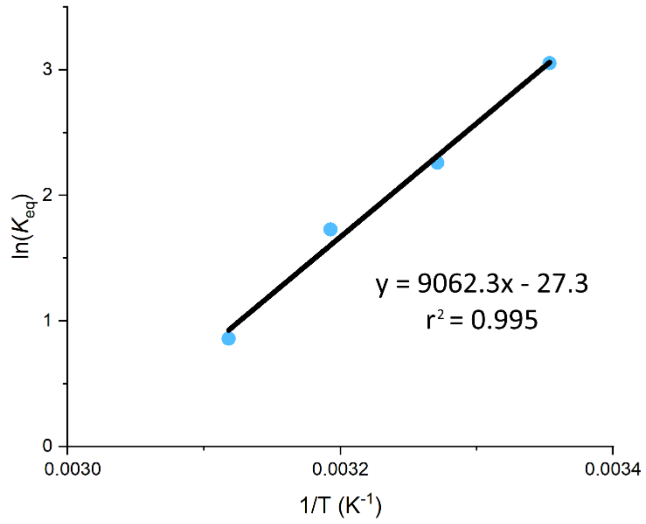


**Figure 1.24** van 't Hoff plot for the conversion of **CYCLE-mono** to **CAT-mono** at [**L-mono**] = 15 mM as a function of temperature.

$$\Delta H = -8.314 \times slope = -75.3\ kJ\ mol^{-1}$$

$$\Delta S = 8.314 \times intercept = -227\ J\ K^{-1}\ mol^{-1}$$

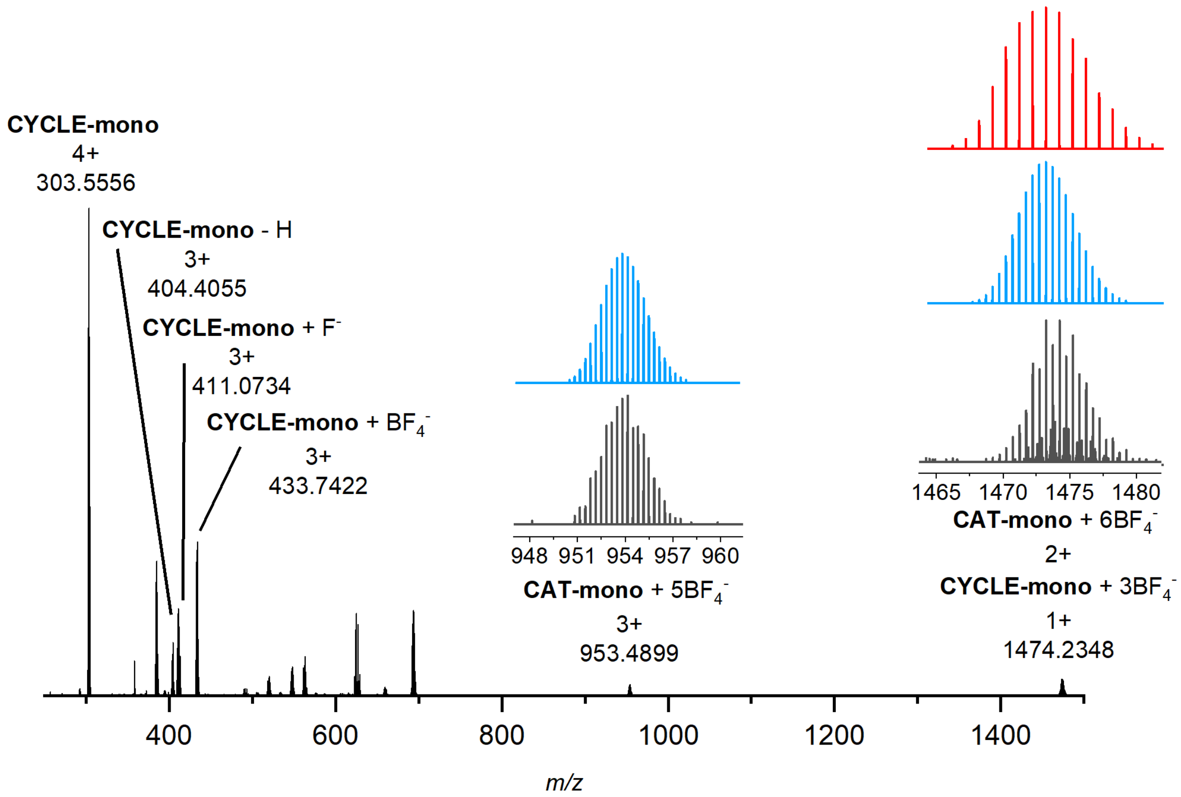


**Figure 1.25** Mass spectrum (DMSO/DMF) of **CAT-mono**. Observed in black, calculated in blue (or red for **CYCLE-mono**).

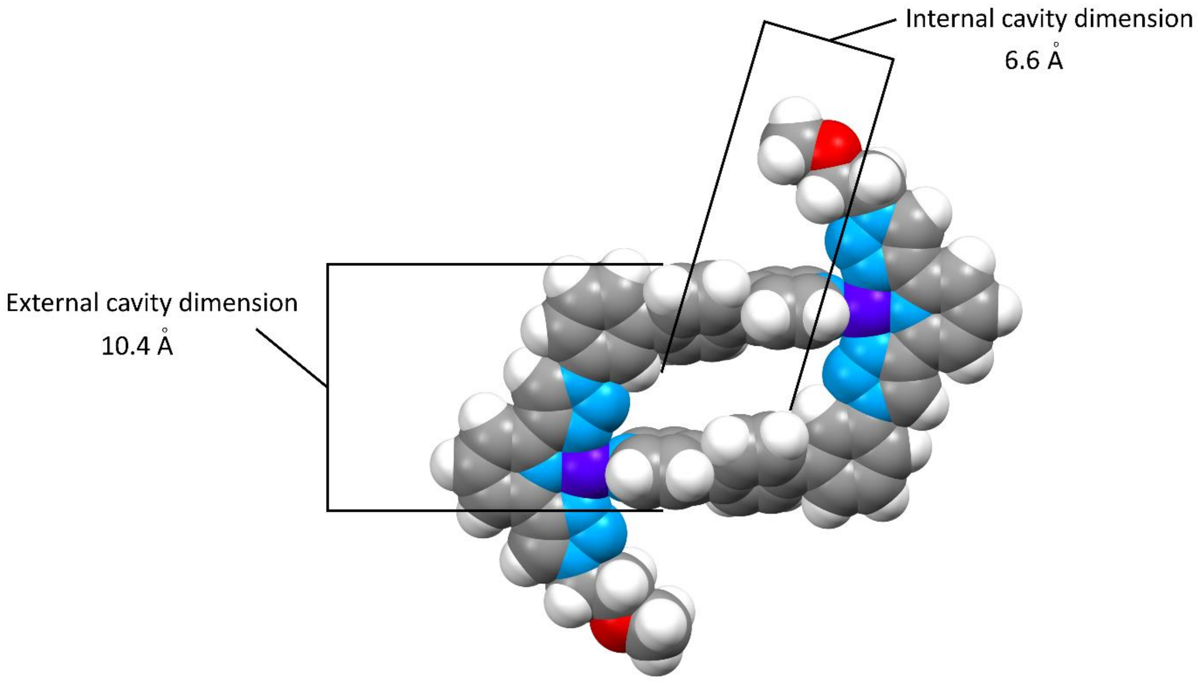


**Figure 1.26** Spacefilling depiction of calculated structure of **CYCLE-mono**, showing internal and external cavity dimensions, which preclude the formation of a ring-in-ring structure. Colours: Carbon grey, nitrogen light blue, palladium dark blue, oxygen red.

### 1.5.3. DUAL-4PEG

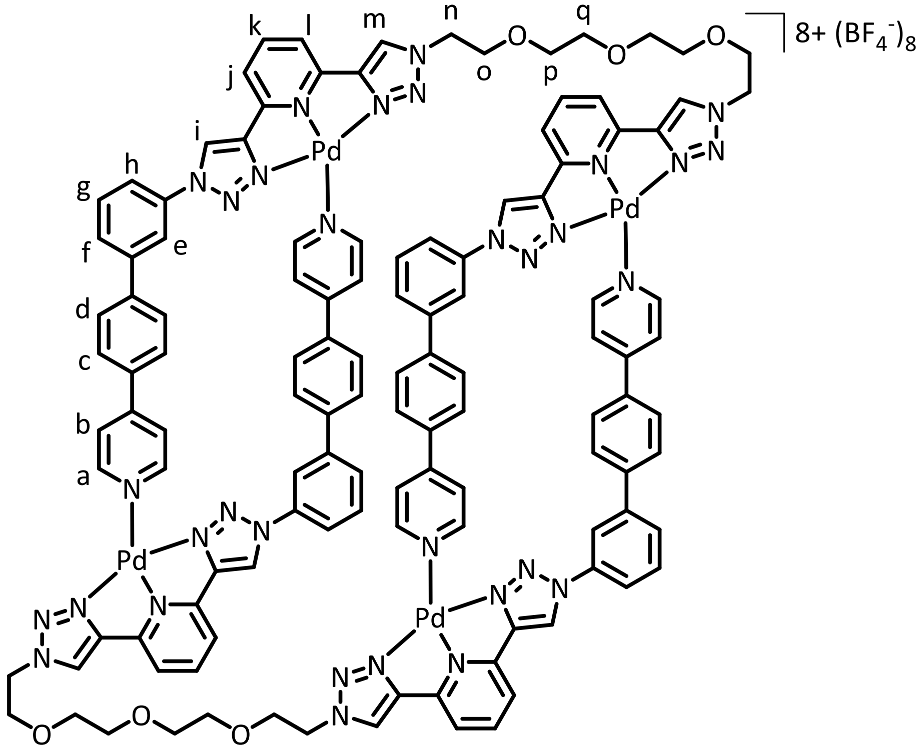


The combination of **L-4PEG** (5.18 mg, 4.96 µmol) and $[Pd(CH_3CN)_4](BF_4)_2$ (4.41 mg, 9.92 µmol) from stock solutions in [D$_6$]DMSO (500 µL) gave quantitative solution phase conversion into the product, [Pd$_4$(**L-4PEG**)$_2$](BF$_4$)$_8$ (**DUAL-4PEG**). Addition of acetonitrile and vapour diffusion of diethyl ether gave

the product as a tan solid. Yield 5.51 mg (68%). $^1$H NMR (400 MHz, [D$_6$]DMSO, 298 K) δ: *integration given per ligand* 10.18 (s, 2H, $H_i$), 9.36 (s, 2H, $H_m$), 8.72 (d, *J* = 6.6 Hz, 4H, $H_a$), 8.68 (t, *J* = 7.8 Hz, 2H, $H_k$), 8.30 (d, *J* = 8.9 Hz, 2H, $H_j$), 8.11 (d, *J* = 8.8 Hz, 2H, $H_l$), 8.08 – 8.07 (m, 6H, $H_b$, $H_e$), 7.83 – 7.75 (m, 8H, $H_c$, $H_f$, $H_h$), 7.70 – 7.66 (m, 6H, $H_d$, $H_g$), 4.94 (br, 4H, $H_n$), 4.10 (br, 4H, $H_o$), 3.90 – 3.64 (m, 8H, $H_p$, $H_q$). $^1$H DOSY NMR *D* (x10$^{-10}$ m$^2$ s$^{-1}$): 0.63. HR ESI-MS (DMSO/DMF) *m/z* = 313.8082 [**DUAL-4PEG**]$^{8+}$ (calc. for [$Pd_4(C_{60}H_{50}N_{16}O_3)_2$], 313.8087); 361.3521 [**DUAL-4PEG** + $BF_4^-$]$^{7+}$ (calc. for [$Pd_4(C_{64}H_{58}N_{16}O_5)_2$]$BF_4$, 361.3560); 425.0773 [**DUAL-4PEG** + 2$BF_4^-$]$^{6+}$ (calc. for [$Pd_4(C_{64}H_{58}N_{16}O_5)_2$]$(BF_4)_{4,}$ 425.0778); 554.2959 [**DUAL-4PEG** + 3$BF_4^-$]$^{5+}$ (calc. for [$Pd_4(C_{64}H_{58}N_{16}O_5)_2$]$(BF_4)_3$, 554.2949); 714.6211 [**DUAL-4PEG** + 4$BF_4^-$]$^{4+}$ (calc. for [$Pd_4(C_{64}H_{58}N_{16}O_5)_2$]$(BF_4)_4$, 714.6215). IR *v* (cm$^{-1}$) 3121, 1618, 1607, 1478, 1290, 1047, 1034, 816.

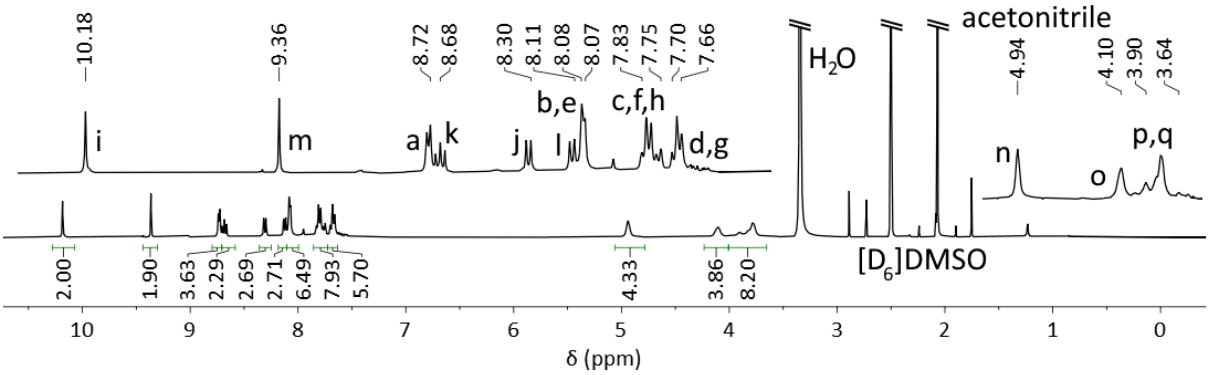


**Figure 1.27** $^1$H NMR spectrum (400 MHz, [D$_6$]DMSO, 298 K) of **DUAL-4PEG**.

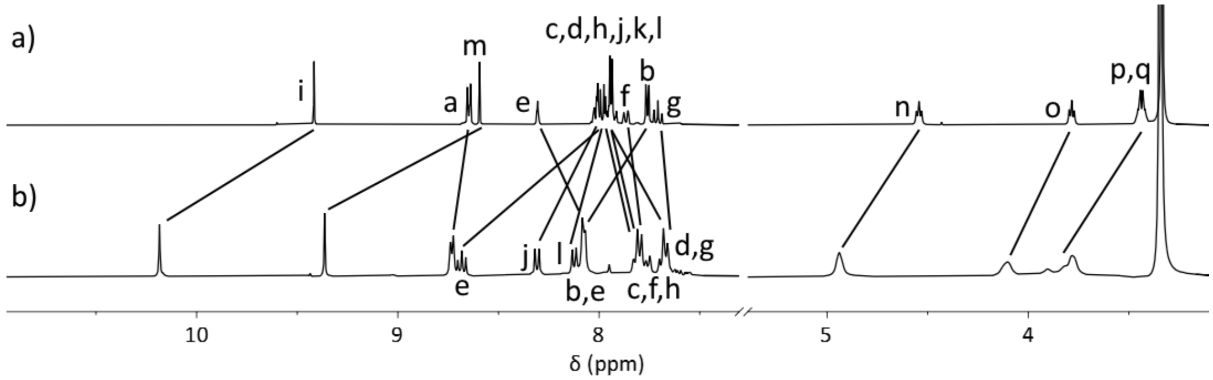


**Figure 1.28** Partial stacked $^1$H NMR spectra (400 MHz, [D$_6$]DMSO, 298 K) of a) **L-4PEG** and b) **DUAL-4PEG**.

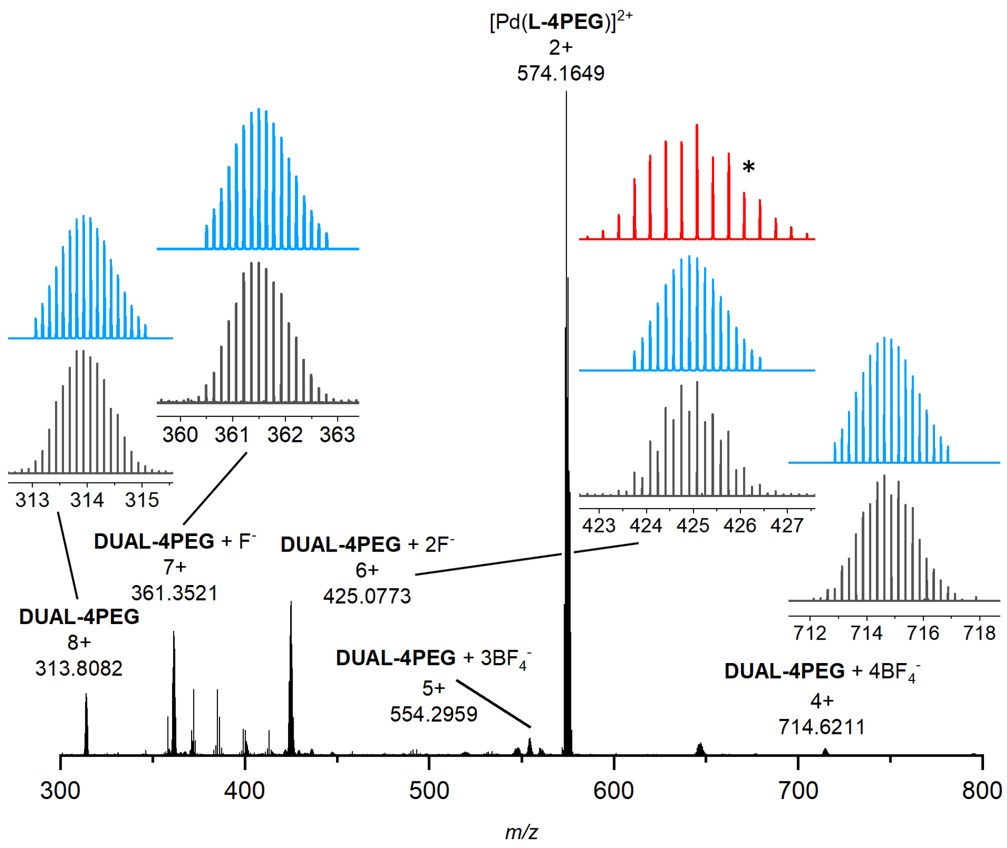


**Figure 1.29** Mass spectrum (DMSO/DMF) of **DUAL-4PEG**. Observed in black, calculated in blue. The calculated isotopic distribution shown in red (*) is derived from the $[Pd_2$(**L-4PEG**) + F$]^{3+}$ fragment.

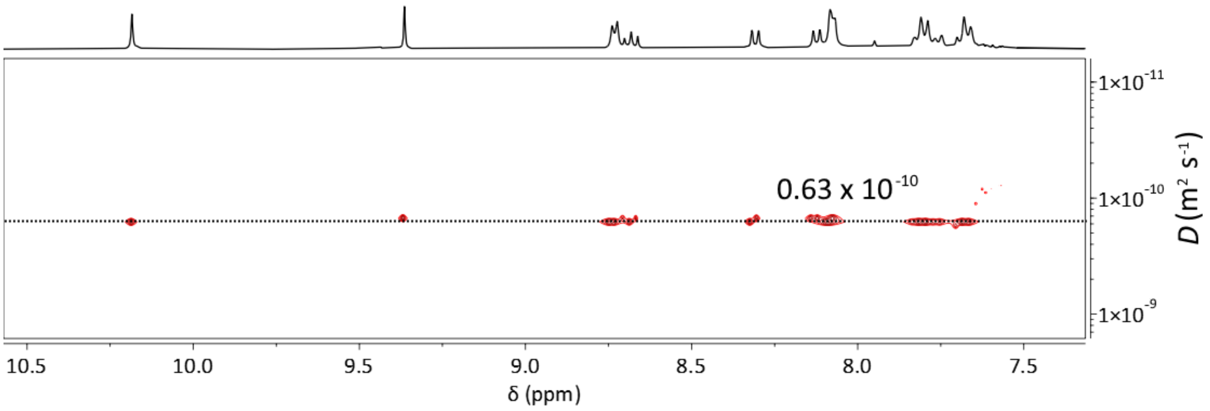


**Figure 1.30** Partial $^{1}$H DOSY NMR spectrum (400 MHz, [$D_6$]DMSO, 298 K) of **DUAL-4PEG**.

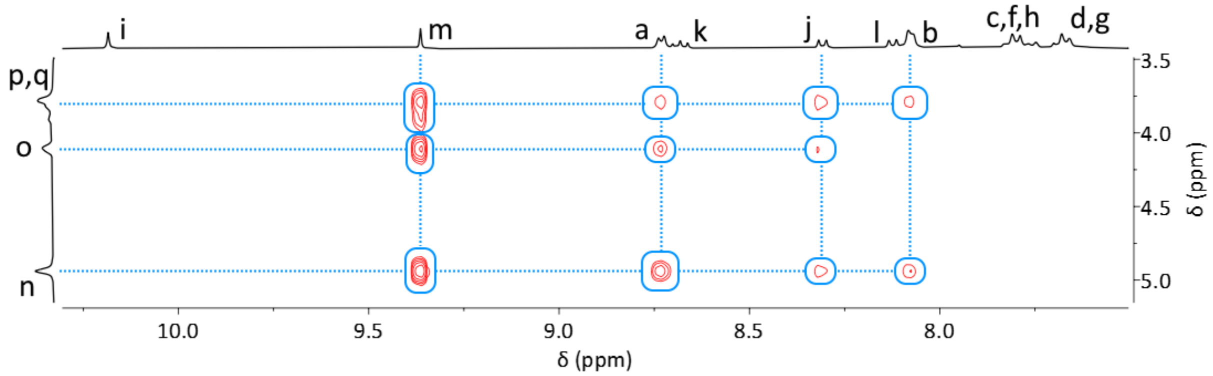

**Figure 1.31** Partial $^{1}H$ NOESY NMR spectrum (400 MHz, [D$_6$]DMSO, 298 K, 200 ms) for **DUAL-4PEG**.

### 1.5.4. TET-6PEG

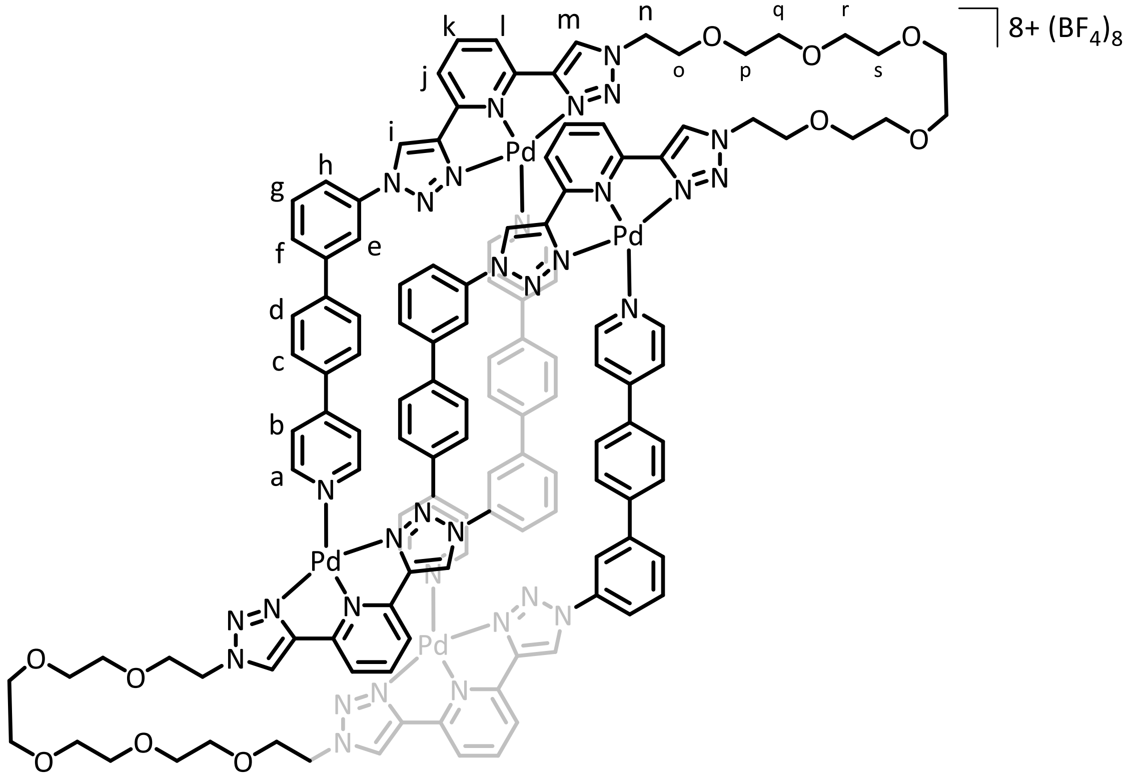

*In proton labelling for this compound, normal labels (e.g. $H_a$ or a) are the 'outer' environment, labels with a prime symbol (e.g. $H_{a'}$ or a') are the 'inner' environment.*

The combination of **L-6PEG** (5.00 mg, 4.42 µmol) and $[Pd(CH_3CN)_4](BF_4)_2$ (3.93 mg, 8.84 µmol) from stock solutions in [D$_6$]DMSO (500 µL) gave solution phase conversion into the product, interlocked [Pd$_4$(**L-6PEG**)$_2$](BF$_4$)$_8$ (**TET-6PEG**), in 85:15 ratio with **CYCLE-PEG** (see below). This compound was analysed solely with solution phase techniques. $^{1}H$ NMR (400 MHz, [D$_6$]DMSO, 298 K) δ: *integration given per ligand, only* **TET-6PEG** *listed for this characterisation* 10.54 (s, 1H, $H_i$), 10.19 (s, 1H, $H_{i'}$), 9.55 (s, 1H, $H_m$), 9.46 (s, 1H, $H_{m'}$), 8.85 – 8.78 (m, 5H, $H_a$, $H_{a'}$, $H_k$), 8.52 – 8.50 (m, 2H, $H_e$, $H_l$), 8.42 (d, $J$ = 7.8 Hz, 1H, $H_j$), 8.32 – 8.27 (m, 3H, $H_h$, $H_{k'}$, $H_{l'}$), 8.12 – 7.55 (m, 17H, $H_b$, $H_{b'}$, $H_c$, $H_{c'}$, $H_d$, $H_{d'}$, $H_{e'}$, $H_f$, $H_{f'}$, $H_g$, $H_{g'}$), 7.42 (d, $J$ = 4.5 Hz, 1H, $H_{j'}$), 6.95 (d, $J$ = 7.8 Hz, 1H, $H_{h'}$), 4.88 (br, 4H, $H_n$, $H_{n'}$), 4.00 (br, 4H, $H_o$, $H_{o'}$), 3.77 – 3.49 (m, 16H, $H_p$, $H_{p'}$, $H_q$, $H_{q'}$, $H_r$, $H_{r'}$, $H_s$, $H_{s'}$). $D$ (x10$^{-10}$ m$^2$ s$^{-1}$): 0.67. HR ESI-MS (DMSO/DMF) $m/z$ = 589.7170 [**TET-6PEG** + 3BF$_4^-$]$^{5+}$ (calc. for [Pd$_4$(C$_{64}$H$_{58}$N$_{16}$O$_5$)$_2$](BF$_4$)$_3$, 589.7190); 758.6472 [**TET-6PEG** +

$4BF_4^{-}]^{4+}$ (calc. for $[Pd_4(C_{64}H_{58}N_{16}O_5)_2](BF_4)_4$, 758.6478); 1040.8655 [**TET-6PEG** + $5BF_4^{-}]^{3+}$ (calc. for $[Pd_4(C_{64}H_{58}N_{16}O_5)_2](BF_4)_5$, 1040.8650); 1604.3005 [**TET-6PEG** + $6BF_4^{-}]^{2+}$ (calc. for $[Pd_4(C_{64}H_{58}N_{16}O_5)_2](BF_4)_6$, 1604.2997).

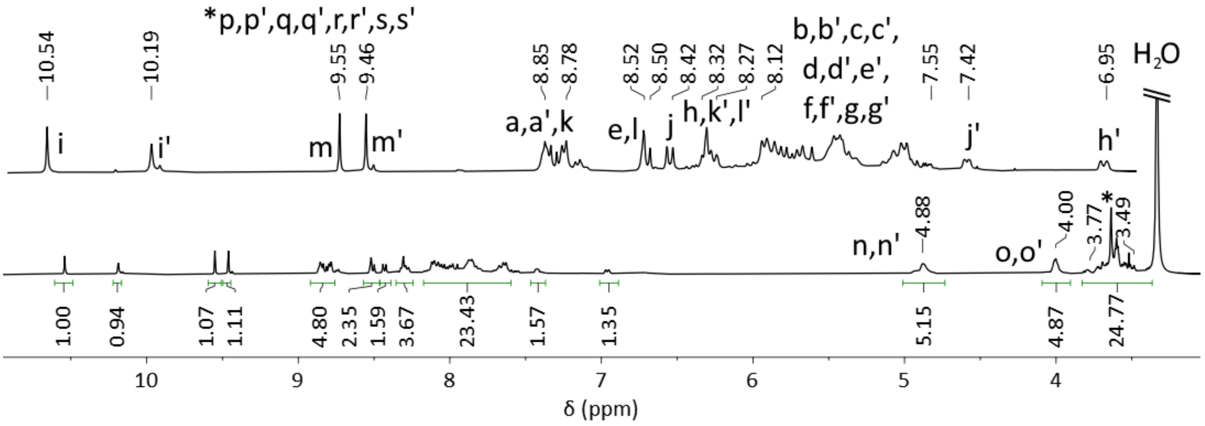


**Figure 1.32** $^1H$ NMR spectrum (400 MHz, $[D_6]DMSO$, 298 K) of **TET-6PEG**.

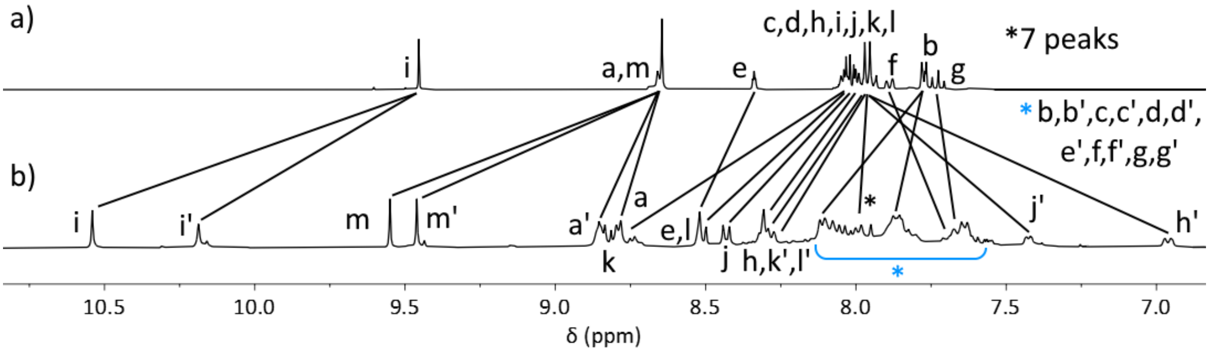


**Figure 1.33** Partial stacked $^1H$ NMR spectra (400 MHz, $[D_6]DMSO$, 298 K) of a) **L-6PEG** and b) **TET-6PEG**.

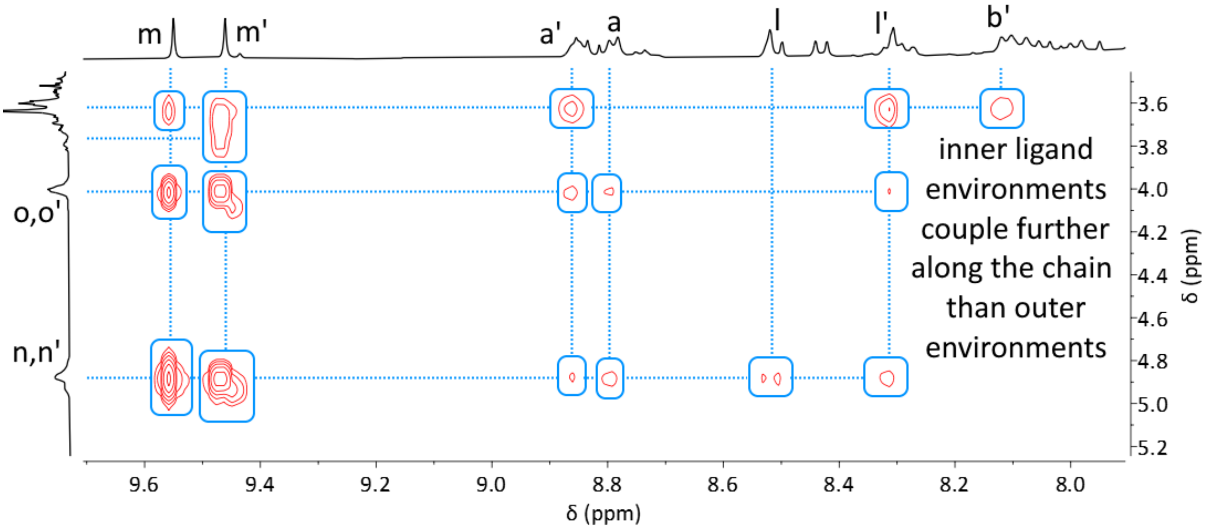


**Figure 1.34** Partial $^1H$ NOESY NMR spectrum (400 MHz, $[D_6]DMSO$, 298 K, 200 ms) for couplings between the aromatic and alkyl regions of **TET-6PEG**.

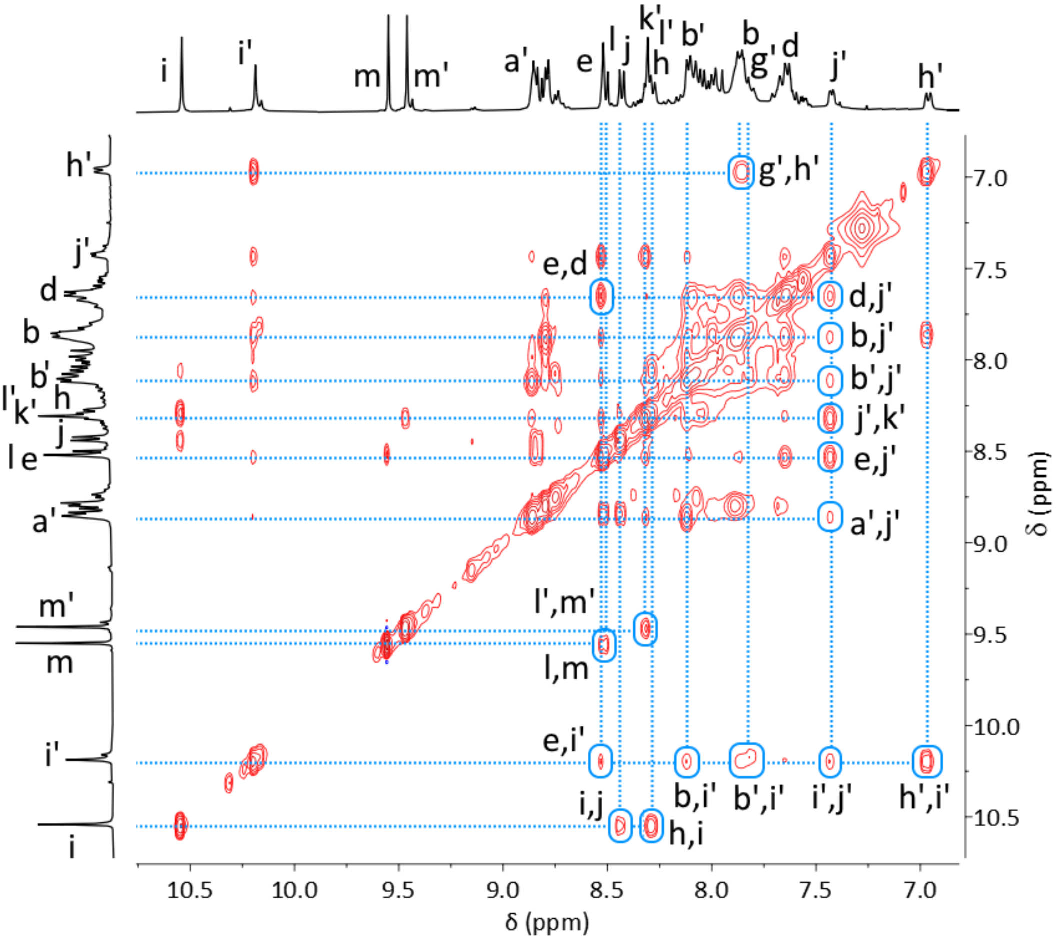


**Figure 1.35** Partial $^{1}H$ NOESY NMR spectrum (400 MHz, $[D_6]$DMSO, 298 K, 200 ms) for the aromatic region of **TET-6PEG**.

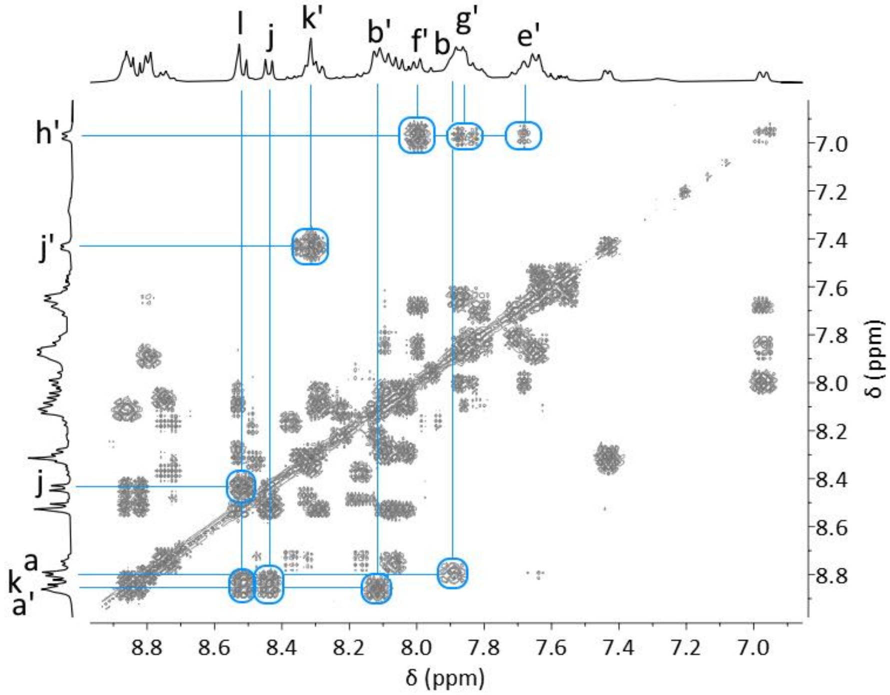


**Figure 1.36** Partial $^{1}H$ TOCSY NMR spectrum (400 MHz, $[D_6]$DMSO, 298 K) for the aromatic region of **TET-6PEG**.

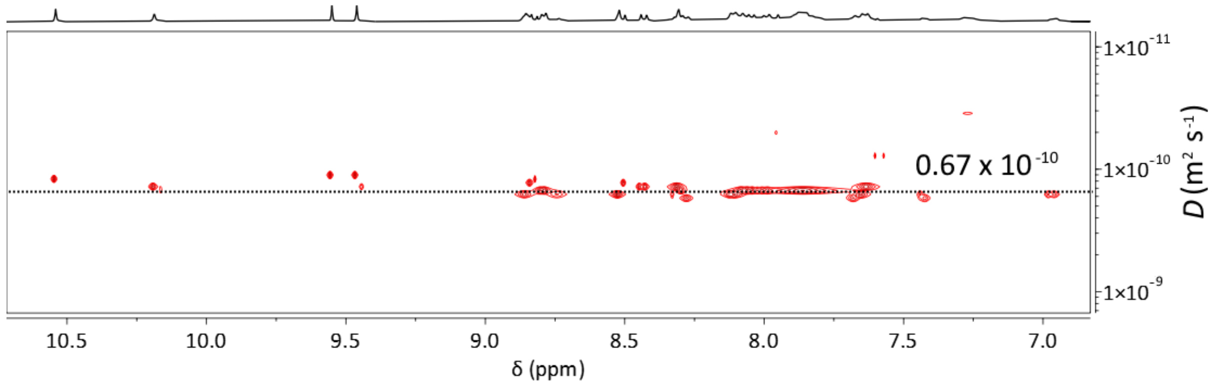


**Figure 1.37** Partial $^1$H DOSY NMR spectrum (400 MHz, [D$_6$]DMSO, 298 K) of **TET-6PEG**.

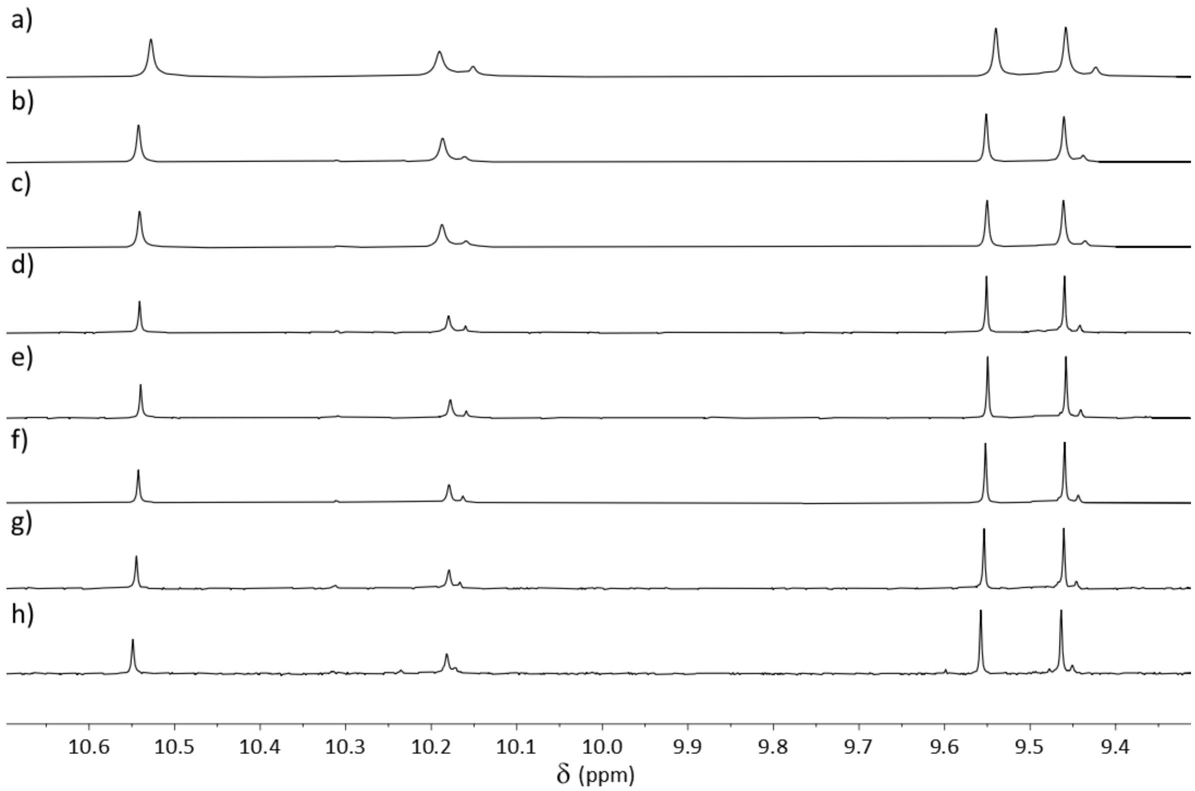


**Figure 1.38** Partial stacked $^1$H NMR spectra (400 MHz, [D$_6$]DMSO, 298 K) of **TET-6PEG** at a) 25 mM, b) 5 mM, c) 2.5 mM, d) 1.0 mM, e) 0.5 mM, f) 0.25 mM, g) 0.125 mM and h) 0.0625 mM, showing no change in ratio to **DUAL-6PEG**.

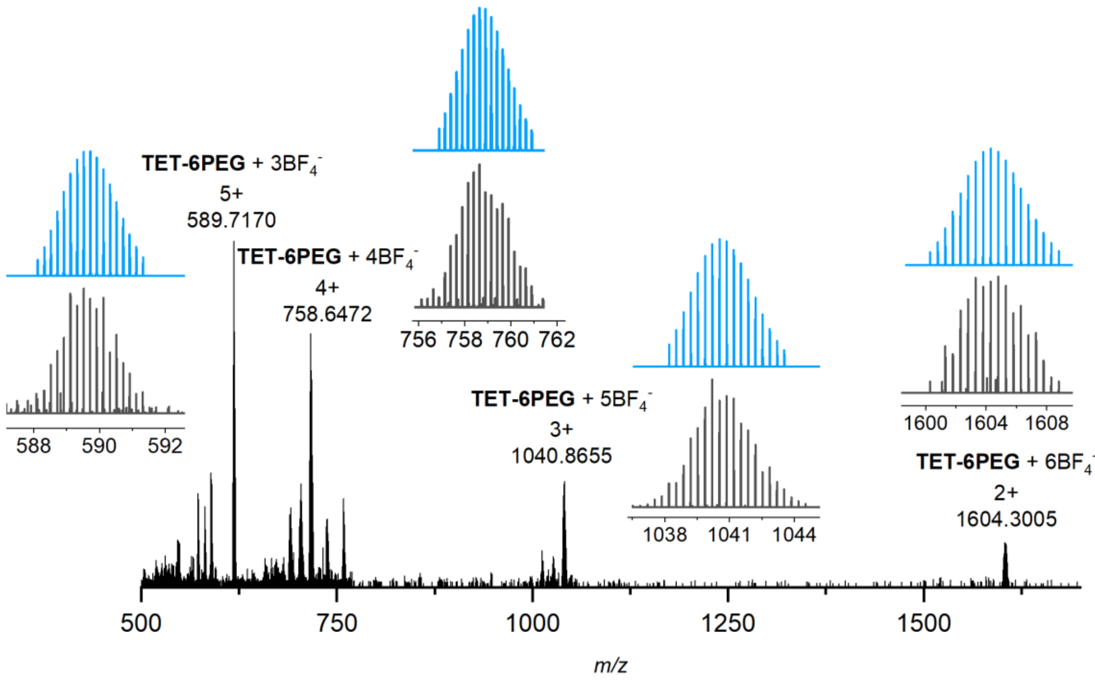


**Figure 1.39** Mass spectrum (DMSO/DMF) of **TET-6PEG**. Observed in black, calculated in blue.

### 1.5.5. DUAL-6PEG

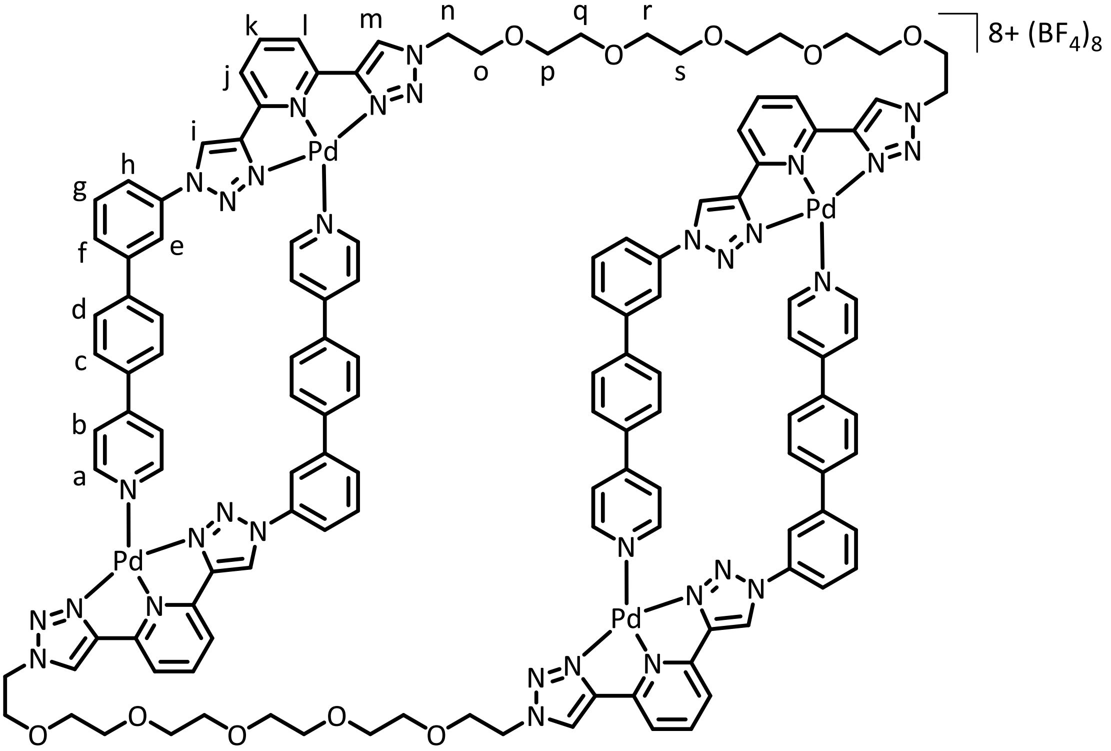


Heating samples of **TET-6PEG** in [D6]DMSO lead to increased conversion into **DUAL-6PEG**. The introduction of other solvents ($CD_3CN$ or $D_2O$) also increased the relative proportion of **DUAL-6PEG**. $^{1}$H NMR (400 MHz, [D6]DMSO, 348 K) δ: *integration given per ligand* 10.10 (s, 2H, $H_i$), 9.47 (s, 2H, $H_m$), 8.87 (d, *J* = 7.0 Hz, 4H, $H_a$), 8.75 (t, *J* = 8.0 Hz, 2H, $H_k$), 8.42 (d, *J* = 7.4 Hz, 2H, $H_j$), 8.24 (d, *J* = 8.2 Hz, 2H, $H_l$), 8.22 (s, 2H, $H_e$), 8.07 (d, *J* = 6.8 Hz, 4H, $H_b$), 7.93 (d, *J* = 7.6 Hz, 2H, $H_h$), 7.89 – 7.80 (m, 10H, $H_c$, $H_d$,

$H_f$), 7.64 (t, $J$ = 6.6 Hz, 2H, $H_g$), 4.97 (t, $J$ = 4.9 Hz, 4H, $H_n$), 4.14 (t, $J$ = 5.0 Hz, 4H, $H_o$), 3.74 (t, $J$ = 5.7 Hz, 4H, $H_p$), 3.67 – 3.64 (m, 4H, $H_q$), 3.61 (m, 8H $H_r$, $H_s$).

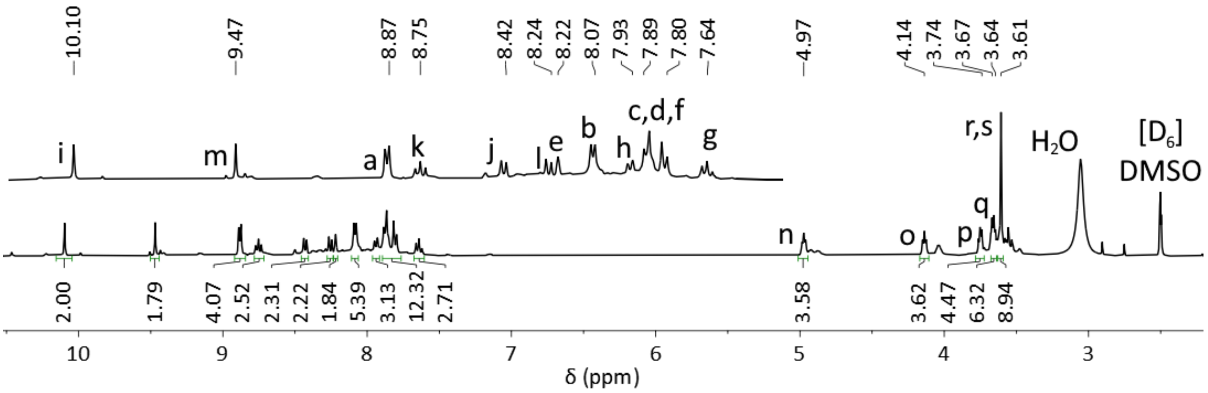


**Figure 1.40** $^1$H NMR spectrum (400 MHz, [D$_6$]DMSO, 348 K) of **DUAL-6PEG**.

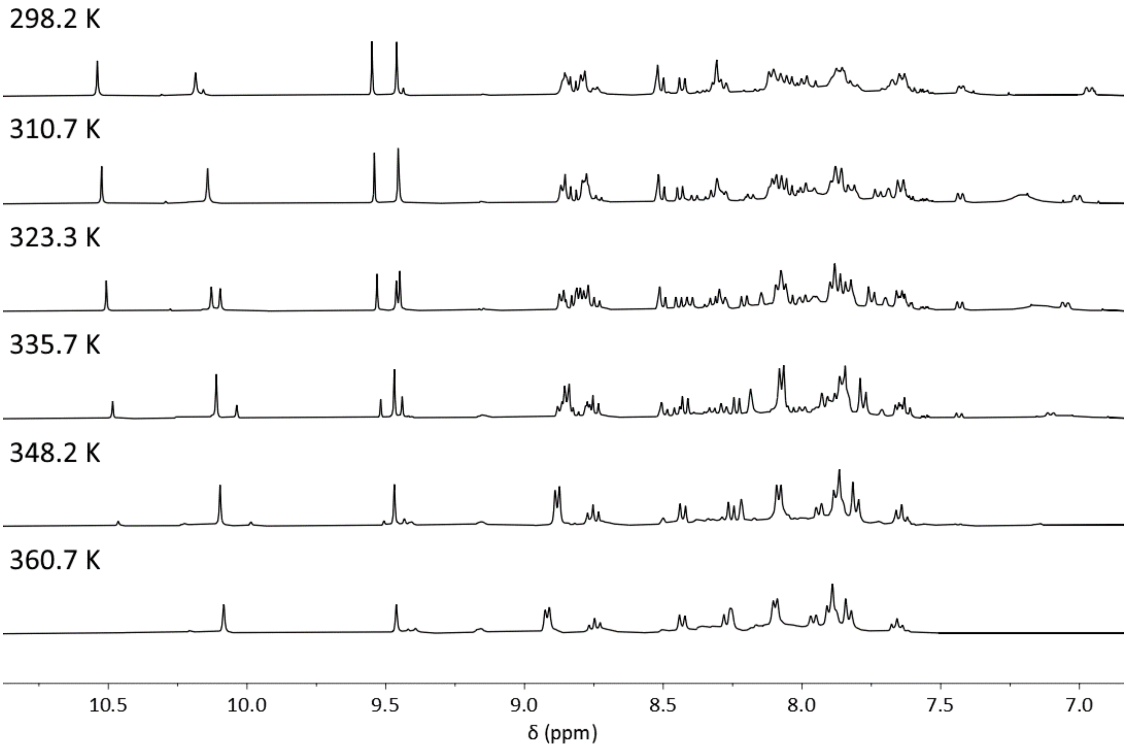


**Figure 1.41** Partial stacked spectra (400 MHz, [D$_6$]DMSO) for the 1:2 combination of **L-6PEG** and Pd(II) at different temperatures. As temperature increases, the proportion of **DUAL-6PEG** relative to **TET-6PEG** decreases.

Expressing the reaction as:

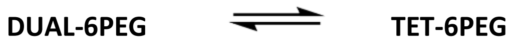


**Table 1.3** Ratios of **DUAL-6PEG** and **TET-6PEG** as a function of temperature, together with $K_{eq}$ at each temperature.

| Temperature (K) | **DUAL-6PEG** | **TET-6PEG** | $K_{eq}$ |
|---|---|---|---|
| 298.2 | 0.163 | 0.837 | 5.1 |
| 310.7 | 0.268 | 0.732 | 2.7 |
| 323.2 | 0.377 | 0.623 | 1.7 |
| 335.7 | 0.564 | 0.436 | 0.8 |

| | | | |
|---|---|---|---|
| 348.2 | 0.752 | 0.248 | 0.3 |
| 360.7 | 0.916 | 0.084 | 0.1 |

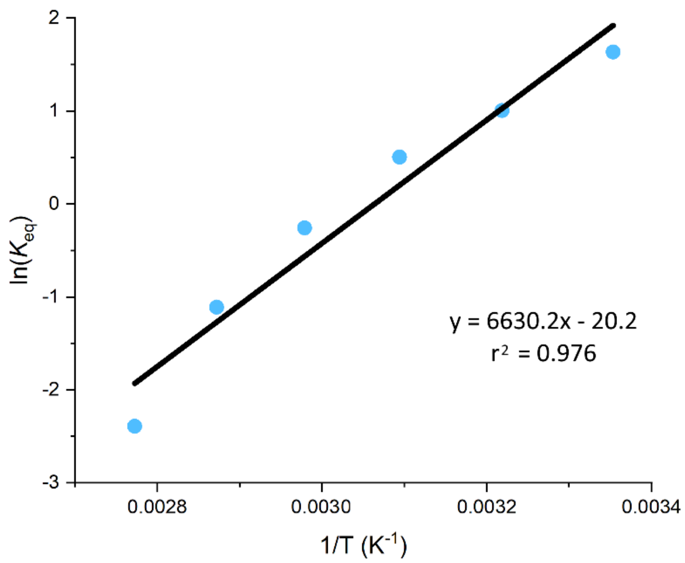


**Figure 1.42** van 't Hoff plot for the conversion of **DUAL-6PEG** to **TET-6PEG** as a function of temperature.

$$\Delta H = -8.314 \times slope = -55.1\ kJ\ mol^{-1}$$

$$\Delta S = 8.314 \times intercept = -167\ J\ K^{-1}\ mol^{-1}$$

## 2. DFT Calculations

Density functional theory (DFT) calculations were performed using the ORCA program version 5.0.[6] Structures were fully optimized using the BP86 functional[7] with C and H atoms treated by the def2-SVP basis set and all other atoms (N, O, Pd) treated by the def2-TZVPP basis set.[8] The resolution of identity approximation was also used with the general auxiliary basis set (def2/J).[9] The Def2-ECP effective core potential was used for Pd[10] and dispersion interactions treated using the D3BJ approach.[11] Structures were optimised with tight convergence criteria on both the geometry and self-consistent field (SCF) cycles. Numerical frequencies were computed to ensure optimised structures represent local minima and to extract vibrational energies. Solvent effects were considered by computing energy with the Conductor-like Polarizable Continuum Model[12] featuring the COSMO[13] epsilon function using the gas phase optimised structures.

As well as providing structural information, we were able to establish that

1. The two possible conformers for **DUAL-6PEG** were similar in energy (3 kJ $mol^{-1}$) and so both would exist, with rapid interchange and/or local environment equivalence on the NMR time scale.
2. A trend exists where as the dielectric constant of the solvent increases (gas phase, acetone, acetonitrile, DMSO) the relative favourability of the ravel with respect to **DUAL-6PEG** increases, in keeping with the experimental data where adding acetonitrile to a DMSO solution of **TET-6PEG** resulted in increased amounts of **DUAL-6PEG**. This is presumably due to enhanced screening of repulsive cationic charge and/or greater solvophobicity for aromatic regions.

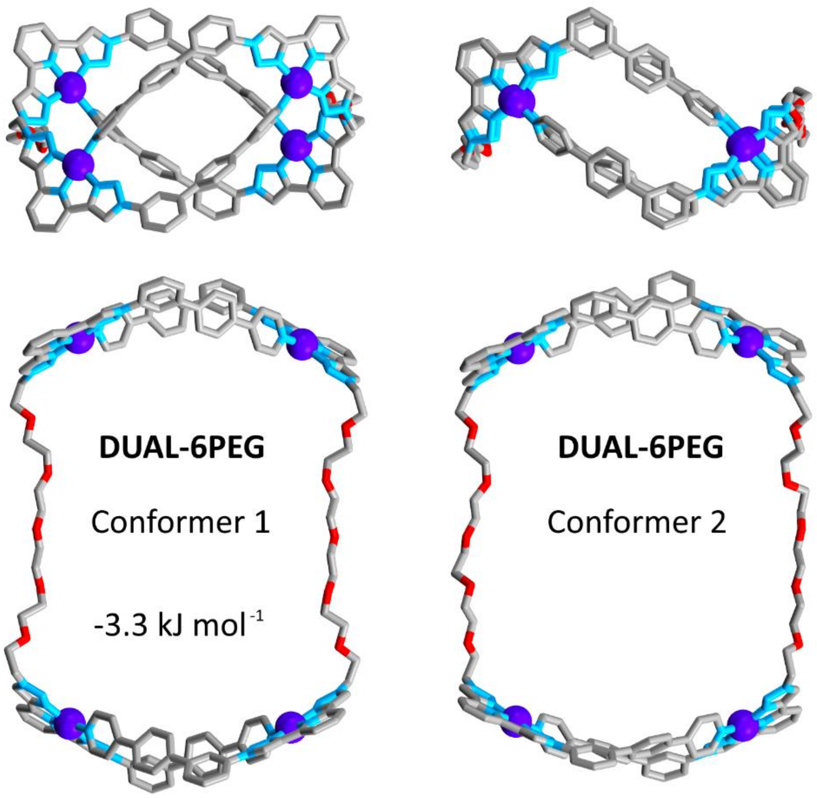


**Figure 2.1** Representations of the two conformers for **DUAL-6PEG**, which are close to energetically equivalent. Colours: Carbon grey, nitrogen light blue, palladium dark blue, oxygen red. Hydrogen atoms excluded for clarity.

**Table 2.1** Simulated Gibbs free energies at 298 K of pertinent species via DFT calculations.

| Products - reactants | Gas phase ΔE ($kJ\ mol^{-1}$) | Acetone ΔE ($kJ\ mol^{-1}$) | Acetonitrile ΔE ($kJ\ mol^{-1}$) | DMSO ΔE ($kJ\ mol^{-1}$) |
|---|---|---|---|---|
| **TET-6PEG** – **DUAL-6PEG**(1) | 429 | -378 | -406 | -414 |
| **DUAL-6PEG**(1) – **DUAL-6PEG**(2) | -3.3 | - | - | - |

# 3. References


[1] Q. V. C. van Hilst, R. A. S. Vasdev, D. Preston, J. A. Findlay, S. Ø. Scottwell, G. I. Giles, H. J. L. Brooks, J. D. Crowley, *Asian Journal of Organic Chemistry* **2019**, *8*, 496-505.

[2] M. Ouchi, Y. Inoue, K. Wada, S. Iketani, T. Hakushi, E. Weber, *J. Org. Chem.* **1987**, *52*, 2420-2427.

[3] Y. Kubo, S. Obara, S. Tokita, *Supramol. Chem.* **2002**, *14*, 171-177.

[4] S. Ito, K. Koizumi, K. Fukuda, N. Kameta, T. Ikeda, T. Oba, K. Hiratani, *Tetrahedron Lett.* **2006**, *47*, 8563-8566.

[5] M. R. Prabhath, J. Romanova, R. J. Curry, S. R. Silva, P. D. Jarowski, *Angew Chem Int Ed Engl* **2015**, *54*, 7949-7953.

[6] F. Neese, *WIREs Computational Molecular Science* **2022**, *n/a*, e1606.

[7] J. P. Perdew, *Physical Review B* **1986**, *33*, 8822-8824.

[8] F. Weigend, R. Ahlrichs, *Physical Chemistry Chemical Physics* **2005**, *7*, 3297-3305.

[9] P. Pollak, F. Weigend, *Journal of Chemical Theory and Computation* **2017**, *13*, 3696-3705.

[10] D. Andrae, U. Häußermann, M. Dolg, H. Stoll, H. Preuß, *Theoretica chimica acta* **1990**, *77*, 123-141.
[11] S. Grimme, S. Ehrlich, L. Goerigk, *Journal of Computational Chemistry* **2011**, *32*, 1456-1465.
[12] V. Barone, M. Cossi, *The Journal of Physical Chemistry A* **1998**, *102*, 1995-2001.
[13] J. Andzelm, C. Kölmel, A. Klamt, *The Journal of Chemical Physics* **1995**, *103*, 9312-9320.
